# Optimizing surveillance post-pandemic: an evaluation of COVID-19 and other respiratory virus surveillance systems in the Philippines, April 2023

**DOI:** 10.1186/s12889-025-24208-8

**Published:** 2025-10-08

**Authors:** Hannah Lofgren, Morgane Donadel, Romel S. Lacson, Devon Ray Pacial, Alethea R. de Guzman, Olivia Almendares, Philippines COVID-19 Surveillance System Evaluation Team, Ma. Gelli Anne Escober, Hanzel Tolentino, Caleb Bongalos, Mark Romel Montevirgen, Kimberly Jane Sotto, Jellrose Mausisa, Phillip P. Salvatore, Sze Man Yiu, Meng-Yu Chen

**Affiliations:** 1 Coronavirus and Other Respiratory Viruses Division, National Center for Immunization and Respiratory Diseases, Centers for Disease Control and Prevention, Atlanta, GA USA; 2 Division of Healthcare Quality and Promotion, National Center for Emerging and Zoonotic Infectious Diseases, Centers for Disease Control and Prevention, Atlanta, GA USA; 3 Epidemiology Bureau, Department of Health, Manila, Philippines; 4 Task Force for Global Health, Atlanta, GA USA; 5 Division of Global HIV and Tuberculosis, Centers for Disease Control and Prevention, Manila, Philippines

**Keywords:** Surveillance, Global health, Cross-Sectional studies, COVID-19

## Abstract

**Background:**

On May 5, 2023, the World Health Organization removed the public health emergency of international concern (PHEIC) designation for the COVID-19 pandemic, noting reduced global risk of severe COVID-19 due to widespread infection and vaccine-induced immunity. Several months prior, the Philippine Department of Health (DOH) requested technical assistance from the U.S. Centers for Disease Control and Prevention (CDC) to evaluate its COVID-19 surveillance ecosystem and inform decision-making for sustainable respiratory virus monitoring. This manuscript describes the evaluation methodology, findings, and recommendations to inform these surveillance transitions.

**Methods:**

In April 2023, an evaluation team from DOH, CDC, and Task Force for Global Health conducted a cross-sectional evaluation of six surveillance systems (COVID-19 case-based, traveler screening, and genomic surveillance; influenza-like illness surveillance, severe acute respiratory infection surveillance, and the Respiratory Syncytial Virus surveillance pilot) at 26 sites across three Philippine regions. Using tailored data collection tools, we analyzed systems’ structure, processes, attributes, and key performance indicators. Findings were categorized into three areas: surveillance system scope and design, system performance, and laboratory capacity.

**Results:**

Although the DOH expanded and upgraded systems for COVID-19 surveillance, including universal case surveillance, contact tracing, and molecular testing and genomic sequencing, rapid development of multiple, isolated systems during the pandemic created inefficiencies and data discrepancies. Prioritizing COVID-19 surveillance strained staff capacity, impacting other surveillance efforts. Key findings included variable reporting completeness and timeliness and underutilization of sentinel sites. Challenges included fragmented data systems, heavy workloads, and resource limitations. Staff adaptability and dedication demonstrated their commitment to surveillance compliance. Progress in molecular testing and genomic sequencing was notable.

**Conclusions:**

Pandemic-era surveillance of respiratory viruses requires adaptation to the interpandemic period. Recommendations focus on right-sizing COVID-19 surveillance while strengthening other surveillance systems, integrating respiratory virus surveillance systems, enhancing staff capacity, and improving stakeholder coordination. These findings offer valuable insights for transitioning to sustainable respiratory virus surveillance post-pandemic.

**Supplementary Information:**

The online version contains supplementary material available at 10.1186/s12889-025-24208-8.

## Summary

### What is already known on this topic

- Countries globally adapted their surveillance and laboratory approaches, often significantly, in size, scope, and performance to respond to the COVID-19 pandemic emergency.
- With the end of the public health emergency of international concern (PHEIC), countries are reviewing how to transition these modifications into sustainable, integrated surveillance of respiratory viruses.

### What this study adds

- Tools and findings from the Philippines’ multi-system respiratory virus surveillance evaluation offer a case study for countries facing decisions on optimizing surveillance infrastructure and operations post pandemic.

### How this study might affect research, practice or policy

- This evaluation and its recommendations align with current global guidance towards achieving integrated surveillance for effective detection and monitoring of respiratory viruses.

## Introduction

Mid-2023 marked a turning point in the COVID-19 pandemic and public health response. On May 5, 2023, the World Health Organization (WHO) declared the end of COVID-19 as a public health emergency of international concern (PHEIC), reflecting a decreased risk of severe COVID-19 globally [1]. By this time, a significant portion of the global population had some protection against SARS-CoV-2, either through infection or vaccine-induced immunity. Following the emergence of the Omicron variant in 2022, global cases of SARS-CoV-2 surged, resulting in an estimated 8.6 billion infections from November 2021 to December 2022 [2]. Shortly after, coverage with primary COVID-19 vaccination series also expanded rapidly [3]. Alongside the end of the PHEIC, the monthly total reported global COVID-19 cases was 1,909,221 in May 2023, just 2% of the monthly total reported during the peak in January 2022 [4]. Individual behaviors and national policies continued to shift, aligning with this juncture in the pandemic. Within public health, one of the most notable changes was a decrease in universal case reporting to WHO, with 50.4% (118/234) of countries discontinuing regular reporting of case and fatality figures [4]. 

Reflecting concerns about declining reporting of COVID-19 surveillance data amidst response demobilization, the Lancet issued a warning in January 2023 encouraging continued transparency in reporting cases, hospitalizations, and deaths, as well as “collaborative surveillance of variant testing.” [5] In 2022, WHO issued revised interim guidance for integrating SARS-CoV-2 surveillance into influenza sentinel surveillance systems [6]. In 2023, further guidance promoted sustainable respiratory surveillance through the Mosaic Respiratory Surveillance Framework [7] and the collaborative surveillance component of the WHO Global Architecture for Health Emergency Preparedness, Response, and Resilience (HEPR) [8, 9]. However, countries continued to grapple with transitioning from emergency response to sustainable monitoring of COVID-19 while addressing disruptions to other health priorities.

The Philippines encountered similar challenges, having invested significantly in adapting existing viral respiratory surveillance infrastructure and developing new approaches to respond to the COVID-19 pandemic. By late 2022, the Philippine Department of Health (DOH) had initiated development of a comprehensive respiratory virus surveillance approach as part of a broader departmental reorganization. Within this context, the DOH’s Epidemiology Bureau (EB) requested technical assistance from the U.S. Centers for Disease Control and Prevention (CDC) to evaluate its COVID-19 surveillance ecosystem and inform decision-making in establishing sustainable and collaborative surveillance for respiratory viruses. The EB also sought to use this opportunity to build local capacity for public health surveillance system evaluation.

This manuscript outlines the methodology used in the 2023 evaluation of COVID-19 and other respiratory virus surveillance systems in the Philippines. It summarizes key findings and recommendations aimed at facilitating the transition from emergency response to sustainable surveillance, particularly for COVID-19 and other respiratory viruses. Presented as a case study, the manuscript emphasizes evaluation tools and insights that may be relevant to countries navigating similar decisions to optimize surveillance infrastructure and operations in the aftermath of the COVID-19 pandemic.

## Methods

### Evaluation

This was a comprehensive cross-sectional evaluation of the Philippines’ COVID-19 and other respiratory virus surveillance systems across national, regional, and local levels. It used a mixed-methods (qualitative and quantitative) approach to assess operations, interconnectivity, and performance, identifying opportunities for improvement focused on systems and practices most useful for sustainable, multisource COVID-19 and other respiratory virus surveillance. Overarching objectives included: (1) describe the structure and processes of COVID-19 and other respiratory virus surveillance systems at all administrative levels; (2) assess key attributes related to user experience including usefulness, acceptability, flexibility, and challenges; (3) assess key performance indicators including data completeness, representativeness, timeliness and overall quality; and (4) evaluate utility of COVID-19 and other respiratory virus surveillance data in describing the person, place, and time of respiratory virus transmission.

In April 2023, an evaluation team comprising 11 program management and epidemiology personnel from the EB, CDC, and the Task Force for Global Health (TFGH) evaluated six Philippine surveillance systems: COVID-19 case-based surveillance, COVID-19 traveler screening, SARS-CoV-2 genomic surveillance, Influenza Like Illness (ILI) surveillance, Severe Acute Respiratory Infection (SARI) surveillance, and the Respiratory Syncytial Virus (RSV) case-based surveillance pilot. Evaluation sites in three of the Philippines’ 17 regions [Cordillera Administrative Region (CAR), Eastern Visayas (EV), and National Capital Region (NCR)] were selected purposively based on willingness to participate, local staff availability, geographic and administrative level diversity, and logistical feasibility for evaluation staff and time allotted to the evaluation. The three regions from which sites were selected also represented two of the three major island groups of the Philippines. Selected sites included mountainous, island, and urban areas, with varying surveillance capacities at different administrative levels (national, regional, provincial, city/municipal), hospitals (public and private), health centers, and laboratories (Additional Table 1). Selected healthcare facilities included ILI sentinel surveillance sites (*n* = 4), SARI sentinel surveillance sites (*n* = 3), and an RSV sentinel surveillance site (*n* = 1), all of which are denoted in Additional Table 1.

### Data collection

To address the evaluation objectives, the CDC team adapted existing surveillance system evaluation guidance and tools [10, 11] to develop three standardized data collection tools tailored to the Philippines context: a tool to document surveillance data structure, process, and flow (Additional File 1); a qualitative interview guide (Additional File 2); and a quantitative Excel-based data extraction tool (Additional File 3). These tools targeted all administrative levels and were used to collect data from key informants working in surveillance, laboratory, and information technology. Prior to data collection, the CDC team conducted a review of grey literature provided by the EB (e.g., manuals of procedures, administrative orders) and virtually introduced EB staff to public health surveillance system evaluation implementation. During the evaluation, the team routinely divided into two groups, each comprised of EB plus CDC and/or TFGH staff, to complete data collection at all sites and offer continuous mentorship to EB staff.

The data structure, process, and flow questionnaire collected information about current surveillance operations at each site, including site structure, processes, specimen and data flows, and inputs and outputs of surveillance systems. Synthesized information documented similarities and differences in operations, specimen testing algorithms, and specimen and surveillance data flows across sites. The qualitative interview guide gathered current perspectives on system usefulness and acceptability through interviews and focus group discussions with key stakeholders. Key informants and focus group participants, including surveillance, laboratory, and program staff, were selected purposively in consultation with EB staff to ensure selection of relevant staff at each site Interviews were not recorded. Instead, dedicated notetakers attended each interview.

### Data analysis

An inductive approach to qualitative analysis was conducted to identify themes related to system attributes and challenges faced by the surveillance workforce. Two reviewers reviewed the key informant interview notes: one reviewer focused on national site interviews while the other reviewer focused on sub-national sites and healthcare facilities. Any disagreement between reviewers during analysis was discussed and resolved through consensus. The quantitative tool was used to extract aggregate surveillance data to evaluate performance of the six surveillance systems. The quantitative analysis had two objectives achieved through review of records from two time periods: (1) retrospective record extraction focused on observations from February 26-April 1, 2023 (epidemiologic weeks 9–13) to evaluate reporting and variable completeness as well as timeliness and quality, while (2) weekly case counts by age group were recorded from July 2021 (or start of system) to March 2023 to account for COVID-19 variant-related surges in evaluation of geographic coverage and representativeness and descriptive epidemiology. Multiple indicators of data quality were evaluated including: reporting completeness (the proportion of sites reporting cases, including zero reporting, during the most recent reporting period over the total number of reporting sites); key variable completeness; and timeliness from symptom onset to specimen collection, from specimen collection to laboratory receipt, and from symptom onset to results reported to public health authority. Disease incidence was also visualized by region. All qualitative and quantitative analyses were conducted using Microsoft Excel v2302 and QGIS 3.28.2.

### Ethics

This evaluation was determined to be public health program evaluation according to the U.S. CDC’s and the Philippine DOH’s human subjects procedures. The evaluation was conducted in accordance with local laws and standards as well as applicable federal law and CDC policy as defined in 45 CRF 46.102(I) [12]. 

## Results

From April 17–26, 2023, we conducted 26 site visits to 14 public health offices and epidemiology and surveillance units (ESUs) and 12 hospitals and health centers/units (collectively referred to as Disease Reporting Units [DRUs]) at national, regional, and local levels across three regions (CAR, EV, NCR) (Additional Table 1). Findings are categorized into three areas: (1) surveillance and information systems scope and design, (2) system performance, and (3) laboratory capacity.

### Surveillance and information systems scope and design

In response to the COVID-19 pandemic, the Philippine DOH implemented new surveillance systems and expanded existing systems (Table 1). The first COVID-19 case in the Philippines, reported on January 30, 2020 in a foreign tourist [13] prompted the DOH’s Bureau of Quarantine (BOQ) to scale up the International Health Surveillance program and accompanying Quarantine Services and International Health Surveillance System (QSIHSS) to detect imported COVID-19 cases for isolation. After declaring a State of Public Health Emergency in March 2020, the Philippines activated the Inter-Agency Task Force for the Management of Emerging Infectious Diseases to guide the government response to the pandemic. To manage the increasing number of cases reported, the DOH established the COVID-19 Surveillance and Quick Action Unit, which implemented universal case-based surveillance, contact tracing, and daily reporting and analysis to meet large-scale COVID-19 surveillance and response needs [14]. To support these activities, two databases were developed with partner assistance, both owned by the EB. COVIDKaya was developed for case-based reporting of epidemiologic data and COVID-19 Document Repository System (CDRS) was developed to maintain a linelist of laboratory records. During the April 2023 surveillance evaluation, universal COVID-19 surveillance and contact tracing remained in place, and BOQ continued to screen passengers on all inbound international flights at Ninoy Aquino International Airport (NAIA) for COVID-19; passenger volumes in April 2023 had returned to 86% of their April 2019 levels [15].

**Table 1:** Respiratory virus surveillance systems evaluated in the Philippines, April 2023

Surveillance System​	Universal COVID-19 Surveillance​			Influenza-Like Illness Surveillance^1^	Severe Acute Respiratory Illness Surveillance^1^​	RSV sentinel Case-based Surveillance Pilot​
	COVID-19 Case-based Surveillance​	COVID-19 Traveler Screening	SCV-2 Genomic Surveillance​			
First line test performed	SCV-2 antigen (at some facilities)​	SCV-2 antigen	4 labs perform SCV-2 WGS​	Quadruplex RT-PCR​ at RITM (Flu A/B, SCV-2, RSV) (sentinel)	Quadruplex RT-PCR at sentinel hospital (Flu A/B, SCV-2, RSV) (sentinel)	Quadruplex RT-PCR at RITM (RSV, Flu A/B)
Second line test performed	SCV-2 RT-PCR (generally performed for all, regardless of antigen test result)	SCV-2 RT-PCR at DOH-BOQ; WGS at RITM (if criteria met)		If RT-PCR neg – 21 target multiplex RT-PCR at RITM	If RT-PCR neg – 21 target multiplex RT-PCR at RITM	none
Individuals eligible for testing	ALL presenting patients who met case definition	Travelers without negative antigen test, with fever or not fully vaccinated	SCV-2 samples verified^2^ by DOH-EB	First 5 patients/week meeting case definition (sentinel)	First 6 patients/week meeting case definition (sentinel)	First 5 patients/week meeting case definition
Geographic Coverage​	national​	Points of Entry​	national​	sentinel case-based (17 sites)​ Nationwide (Passive)​	sentinel case-based (5 sites)​ Nationwide (Passive)	sentinel (3 sites)​
Managing Agency​	DOH-EB​	DOH-BOQ​	RITM, PGC​	RITM (Sentinel),​ DOH-EB (Passive)​	RITM (sentinel),​ DOH-EB (Passive)​	RITM​
Information System​	COVIDKaya (old system)​ TKC (new system – CIF and lab results)​ CDRS (lab results linelist and CIF)​		REDCap→ GISAID​	PIDSR-IS/EDCS-IS (Sentinel & Passive)​	CDRS (sentinel)​ PIDSR/EDCS-IS (sentinel & Passive)​	Excel ​
Reporting Process	RT-PCR results reported to COVIDKaya or TKC and CDRS (antigen tests not routinely reported)			All results reported to PIDSR-IS/EDCS-IS	If SCV-2, treated as COVID-19 patient; sample not sent to RITM (sentinel) All other sentinel results reported to PIDSR-IS/EDCS-IS	Results reported back to DOH-EB and hospital

*RSV* Respiratory Syncytial Virus, *WGS* Whole Genome Sequencing, *rt-PCR* Real-Time reverse transcriptase Polymerase Chain Reaction, *SCV-2* SARS-CoV-2, *RITM* Research Institute for Tropical Medicine, *DOH-EB* Department of Health-Epidemiology Bureau, *DOH-BOQ* Department of Health-Bureau of Quarantine, *PGC* Philippine Genome Center, *GISAID* Global Initiative on Sharing All Influenza Data, *CIF* Case Investigation Form, *TKC* Tanod-Kontra COVID, *CDRS* COVID Data Repository System, *EDCS-IS* Epidemic-prone Disease Case Surveillance—Information System, *PIDSR-IS* Philippines Integrated Disease Surveillance and Response Information System

^1^Refers to both sentinel active case-based and nationwide passive ​surveillance

^2^Eligible samples must have been taken within the last 14 days; originated from an active COVID-19 case; have a Cycle-threshold value ≤31; and a volume of ≥500uL

Increased demand for COVID-19 testing led to expanded investment in molecular testing and genomic sequencing across the Philippines. For example, the number of SARS-COV-2 molecular testing laboratories accredited by the Philippine Health Insurance Corporation (PhilHealth), the nation’s National Health Insurance Program administrator, grew from 114 government and private laboratories in 2020 to 234 total laboratories in 2022 [16, 17]. By 2023, four national laboratories were performing SARS-CoV-2 whole genome sequencing (WGS). The expansion of regional sequencing capacity was led by the EB and supported by the Philippine Genome Center (PGC) and the Genomic Epidemiology of COVID-19 in the Philippines (GECO-PH) initiative, led by the Research Institute for Tropical Medicine-Philippine National Influenza Center (RITM), a DOH research institution designated as the national reference laboratory for influenza and other respiratory viruses. Facilities and DOH Regional ESUs sending specimens to PGC and RITM for sequencing tracked all information in Google Sheets, while RITM maintained a standalone REDCap database [18] of sequenced specimens. Sequencing results and metadata were submitted to the EB where they were analyzed.

In addition to these new and/or modified surveillance approaches, RITM maintained a sentinel surveillance network of 17 outpatient facilities (referred to as ILI sites) and five hospitals (referred to as SARI sites) established prior to the pandemic. An ILI case was defined as having fever (≥ 38 °C) and cough with onset in the past 10 days, and a SARI case was defined as having fever (≥ 38 °C) and cough with onset in the past 10 days requiring hospitalization for difficulty breathing. Specimens collected from a sample of individuals meeting these case definitions at these sentinel sites underwent molecular testing at RITM and/or hospital laboratories to monitor transmission of influenza. Beginning in April 2020, these specimens were also tested for SARS-CoV-2 by reverse transcription-polymerase chain reaction (RT-PCR) [19]. Nationally, non-sentinel sites were requested to report aggregate numbers of cases meeting the ILI case definition to public health authorities, but specimens were not routinely collected from these cases for molecular detection; reporting of *cases* meeting the ILI and SARI case definitions from non-sentinel sites was ad hoc at clinician discretion.

In November 2021, RITM initiated pilot RSV sentinel surveillance in the first of three SARI sentinel sites as part of WHO’s global RSV surveillance pilot for integration into the expanded Global Influenza Surveillance and Response System (eGISRS). This surveillance successfully launched despite pandemic delays, but there was no formal information system for RSV surveillance data, and as a pilot, it operated separately from other respiratory disease surveillance systems. Regular supervisory visits or calls to assess and address performance in any sentinel site were not routine, except in RSV sites.

Based on early pandemic response experience and data needs moving forward, the EB upgraded the information systems for COVID-19 and ILI/SARI surveillance. This paper will refer to COVID-19-surveillance (case-based active reporting via multiple surveillance systems of all cases positive for SARS-CoV-2 regardless of clinical presentation) as universal COVID-19 surveillance, and to ILI/SARI sentinel surveillance (active reporting of cases meeting ILI/SARI case definitions at designated healthcare facilities and tested by RT-PCR), as sentinel surveillance. In early 2023, the TanodKontraCOVID (TKC) information system was created to replace COVIDKaya, whose infrastructure could no longer support the volume of COVID-19 data required. Similarly, the Epidemic-prone Disease Case Surveillance – Information System (EDCS-IS) replaced the Philippines Integrated Disease Surveillance and Response Information System (PIDSR-IS) offline application as the national notifiable disease surveillance information system, which initially included nationwide passive ILI case surveillance and optional passive SARI case surveillance. These upgrades coincided with the evaluation in April 2023 [20] (Table 1), such that users had mixed access to and preferences for the new information systems, with many operating different systems or using both old *and* new systems concurrently. Ultimately, during the COVID-19 pandemic, DOH established new national systems for reporting epidemiological and laboratory data of multiple respiratory pathogens to public health authorities: CDRS (for universal COVID-19 laboratory testing), COVIDKaya and TKC (for universal COVID-19 case investigation and sentinel surveillance), and PIDSR-IS and EDCS-IS (for nationwide passive ILI case surveillance and optional passive SARI case surveillance). Alongside in-house databases unique to specific organizations, eight information systems were in concurrent use for respiratory virus surveillance at the time of the evaluation.

### Surveillance system performance

#### Reporting and key variable completeness

Of the six systems evaluated, data necessary to evaluate record completeness were available from four. Key demographic variables including sex and residence had high completeness (< 1% missing) in these four surveillance systems. Completeness proportions for other key variables were more variable across systems. High completeness of data on age and illness onset, for example, was observed in all four systems except the COVID-19 case-based surveillance, for which 3.9% and 66.0% of cases were missing these data (Table 2). Other systems were observed to have a greater proportion of missing values for other variables; for example, patient outcome was missing for 99.5% of records in the SARI sentinel surveillance network but universally reported in other systems. Laboratory results were missing at higher proportions from the two passive reporting systems (20.7% in SARI and 93.7% in ILI). No missing data were reported for variables from RSV surveillance pilot data (Table 2). Two-thirds of ILI and SARI sentinel sites and COVID-19 case-based reporting sites reported data in the 30 days prior to evaluation site visits, while all RSV sentinel sites reported data during this period (Additional Table 2).

**Table 2:** Proportion missing & unknown values for key demographic and health event variables for COVID-19 case surveillance, sentinel and passive ILI and SARI surveillance at national and/or subnational levels during February 26- April 1, 2023

	COVID-19 Case-based Surveillance	ILI Surveillance		SARI Surveillance		RSV sentinel Surveillance^1^
Key Variable	All reporting sites nationally *N* = 6,533 cases	Passive Surveillance *N* = 15,738 cases	sentinel Surveillance^1^ *N* = 226 cases	Passive Surveillance *N* = 217 cases	sentinel Surveillance^1^ *N* = 217 cases	Three pilot sites *N* = 39 cases
Age	3.9	0.5	0	0	0	0
Sex	0	0	0	0	0.5	0
Residence	0	0	0	0	0.5	0
Vaccination status	NR	NR	NR	NR	21.2	NC
Illness Onset Date	66.0	0.3	0	0	0	0
Specimen Collection Date	0.6	NR	0	66.8	0.9	0
Hospital Admission Date (if hospitalized)	99.9	NC	NC	0	15.2	0
Hospital Discharge Date (if hospitalized)	NR	NC	NC	89.4	100.0	NC
Signs/Symptoms	NR	NR	0	27.6	0	0
Lab test results	0	93.7	NR	20.7	0	NC
Outcome (survived or not)	0	0	NR	0	99.5	NC

*ILI* Influenza-like Illness, *SARI* Severe Acute Respiratory Infection, *RSV* Respiratory Syncytial Virus, *NR* Not reported to the evaluation team, *NC *Not captured on the CIF

^1^These data reported as per completeness at the Research Institute for Tropical Medicine

#### Timeliness

**Fig. 1:**
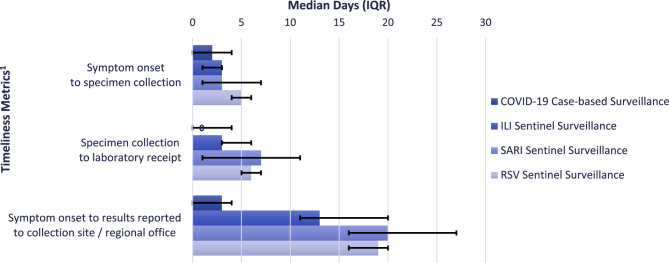
Timeliness between symptom onset/specimen collection and reporting steps for COVID-19, ILI, SARI, and RSV surveillance systems during February 26 – April 1, 2023, Philippines. ILI: Influenza-like Illness, SARI: Severe Acute Respiratory Infection, RSV: Respiratory Syncytial Virus, IQR: Interquartile Range. ^1^Data for ILI, SARI, and RSV sentinel surveillance systems were reported by the Research Institute for Tropical Medicine; COVID-19 case-based surveillance data reported by Philippine DOH. Data from the first five observations (i.e., cases) during each week were used to calculate timeliness metric. The COVID-19 cases metric‘Symptom onset to results reported to collection site/regional office’ refers to the date the case was reported to collection site; for ILI, SARI, and RSV sentinel surveillance, this is the date the case was reported back to regional office

Timeliness of reporting varied by surveillance system. COVID-19 case-based surveillance had the timeliest reporting with a median of 3 days (interquartile range [IQR]: 0,4) from symptom onset to reporting laboratory results back to collection site (Fig. 1). Other respiratory virus surveillance systems had longer median days from symptom onset to regional health office notification: ILI sentinel surveillance (14 days, IQR: 12, 15), SARI sentinel surveillance (20 days, IQR: 16, 27), and RSV surveillance (19 days, IQR: 16, 20). Median days from symptom onset to specimen collection were similar for COVID-19 case-based (2 days) and ILI sentinel (3 days), SARI sentinel (3 days), and RSV sentinel (5 days) surveillance. Median days from specimen collection to laboratory receipt were shorter for universal COVID-19 (0 days) compared to ILI sentinel (3 days), RSV (6 days), and SARI sentinel (7 days) surveillance. Timeliness at regional or facility levels was not evaluated due to fragmented data systems and missing dates of case reports to ESUs.

#### Geographic coverage

**Fig. 2:**
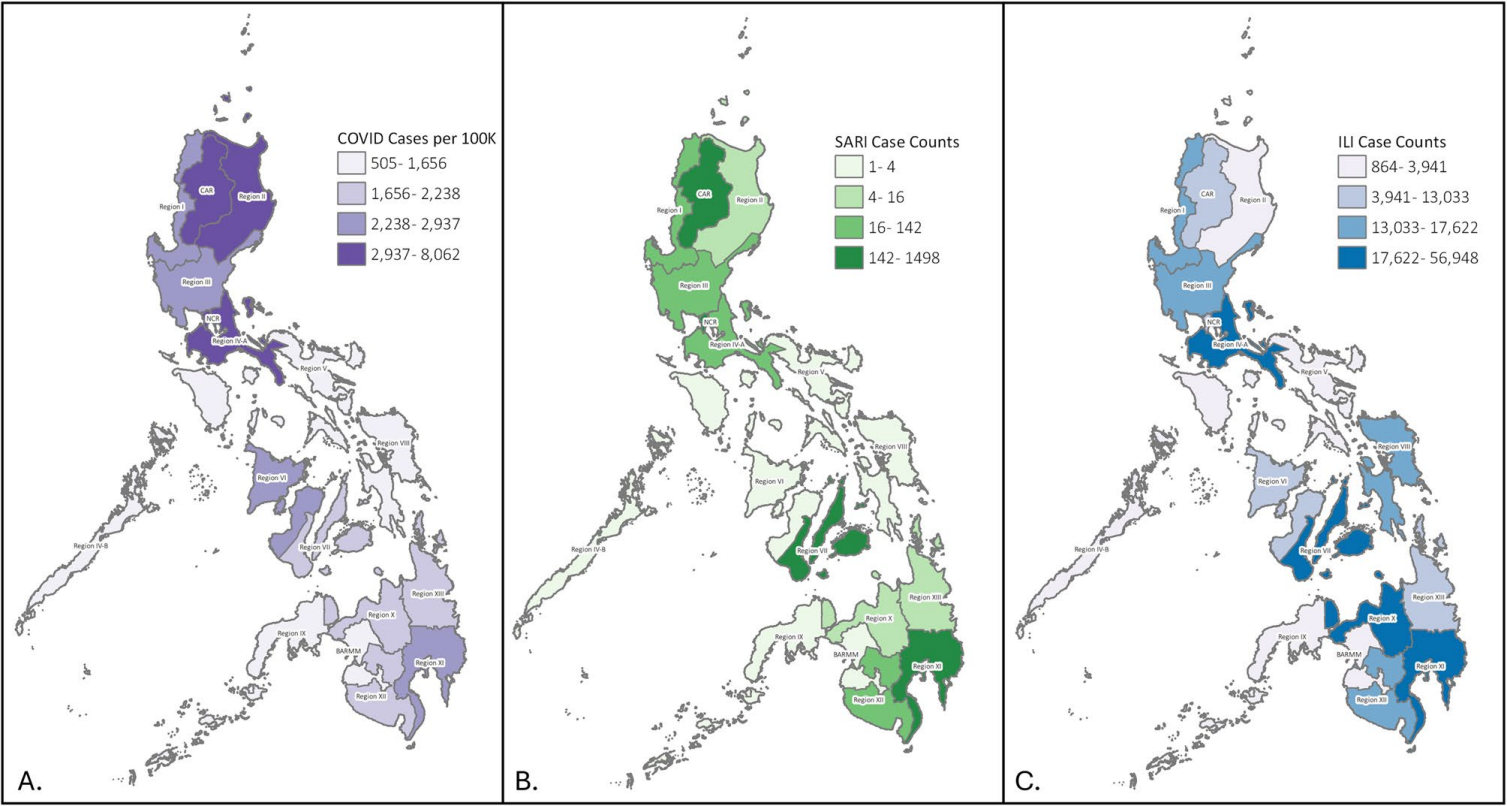
COVID-19 cases per 100,000 population^1^ **A** and ILI **B** and SARI **C** case counts in the Philippines by Region – January 2021 to March 2023^2^. ILI: Influenza-like Illness, SARI: Severe Acute Respiratory Infection. ^1^Data for COVID-19 cases includes cases reported as part of universal COVID-19 surveillance. ^2^These data reflect the cumulative number of cases during the specified time period, for COVID-19 these are standardized by the total population per region; for ILI and SARI these are reported case numbers. Data for ILI and SARI surveillance include passively reported cases from all health centers and hospitals based on whether patients met the respective syndromic case definitions. In regions with sentinel health centers or hospitals, active case reporting is conducted, including laboratory testing of a subset of specimens from reported cases

Sentinel systems each included sites in different regions, and participation in passive surveillance varied by region, making direct comparison of reported case counts and incidence rates across systems challenging. Nonetheless, notable patterns emerged. From January 2021 to March 2023, CAR, NCR, Region II (Cagayan Valley), and Region IV-A (Calabarzon) had the highest COVID-19 case rates per 100,000 population at 8073, 6678, and 4533, respectively (Fig. 2). For ILI surveillance, Regions XI (Davao) and X (Northern Mindanao) reported the highest case counts (56,948 and 35,580), while for SARI surveillance, Region VII (Central Visayas), and CAR reported the most cases (1,498 and 1,196). Rates could not be calculated for ILI and SARI surveillance due to undefined catchment areas and variation in reporting. Notably, regions with high COVID-19 incidence did not consistently align with those reporting high ILI or SARI case counts. For example, CAR had both high COVID-19 incidence and SARI cases while Regions VII and XI reported high SARI and ILI cases —despite lower COVID-19 rates. These differences likely reflect variation in surveillance intensity and sentinel site distribution. Regions with high SARI case counts typically had active surveillance and specimen collection, whereas regions with high ILI counts relied more on passive surveillance. While these comparisons provide useful descriptive insights, they do not represent direct comparisons of disease burden, as ILI and SARI case data cannot be standardized to population-level incidence rates.

#### Data reporting and information management

During key informant interviews, surveillance and laboratory staff expressed challenges with surveillance data reporting and information management, citing few linkages between epidemiologic and laboratory information systems (e.g., COVIDKaya and CDRS) and a lack of interoperability among multiple new and existing systems (e.g., PIDSR-IS to EDCS-IS and COVIDKaya to TKC). Additionally, staff noted complex user activation processes for new systems like TKC, coupled with the inability to access historic data after transition to a new system. Staff noted that all of these challenges led to duplicative workflows.

Regional and local level staff stressed the importance of their input in designing information systems and surveillance tools. Some DRUs reported minimal or no feedback from the national level and recurrent discrepancies between national and regional or local data. Key informant interviews also revealed that information systems did not meet end user needs, resulting in the maintenance of site-specific linelists, implementation of “workarounds” to meet local data and analysis requirements, and low confidence in national level data quality. Staff reported using Google Sheets, Excel spreadsheets, or WhatsApp/Viber/Facebook messages for reporting COVID-19 surveillance data. At the national level, staff indicated that surveillance data reporting lacked standardization, leading to increased data management challenges.

#### Human resources

Healthcare workers and surveillance, laboratory, and information technology staff reported heavy workloads from increased surveillance demands to capture all COVID-19 cases, in addition to other respiratory surveillance responsibilities, exacerbated by reporting and information system challenges. Underpinning these difficulties, staff noted limited financial and human resources for surveillance, high staff turnover, insufficient capacity-building opportunities, and unclear job descriptions for surveillance roles. In order to manage the sudden onset of expanded surveillance activities, DOH financed a surge of short-term surveillance officer contracts from 2020 to 2023. At the time of evaluation, surveillance staff expressed concerns about workload sustainability since many of these were short-term contracts. Despite these challenges, key informant interviews suggested that staff at all sites evaluated were committed to complying with surveillance and laboratory reporting requirements.

### Laboratory capacity

#### Testing

Universal COVID-19 surveillance in the Philippines was a “test-all” strategy, and suspect and probable cases were not included in national counts or shared through national surveillance reports. In all healthcare facilities, presenting patients were tested for SARS-CoV-2 if they reported an exposure to a suspected or confirmed COVID-19 case or met COVID-19 symptom criteria. In some facilities, all patients presenting for emergent/inpatient care were first screened using a SARS-CoV-2 antigen test and in at least one evaluated facility, this was done regardless of symptoms, with positive tests confirmed by RT-PCR. Diagnosis without a confirmatory test result was not noted as a component of surveillance in any of the evaluation sites. Although antigen testing was common in many sites, results were not uniformly reported. Despite some variation in testing practices, universal COVID-19 surveillance data was valued for decision-making across sites and levels and fostered the expansion of molecular laboratory capacity for SARS-CoV-2.

Laboratories entered test results and updated case investigation forms (CIFs) into CDRS. The process for manual CIF and test result entry into TKC was dependent on the testing location of the patient. Given the ongoing transition from COVIDKaya to TKC, not all users could batch upload linelists of cases into TKC at the time of evaluation, further complicating the digitization process. Regardless of the originating healthcare facility, entry of test results into TKC marked the first notification of a COVID-19 case to national DOH authorities.

At the time of the evaluation, many laboratories tested for SARS-CoV-2 only, due to limited training and resources, as well as challenges in obtaining proper licensure for multiplex testing. Testing for non-SARS-CoV-2 respiratory viruses was primarily conducted at RITM. ILI and RSV sentinel specimens were tested at RITM, while SARI sentinel specimens were tested locally using a quadruplex assay (Influenza A/B, SARS-CoV-2, RSV) and referred to RITM for testing by multiplex PCR if negative for all quadruplex assay targets. RSV cases were screened for SARS-CoV-2 at admission, and further tested by multiplex (Influenza A/B and RSV) PCR at RITM (Table 1). The weekly target for sentinel sample collection (5 for ILI and 6 for SARI) was often not met and delays due to high demand for SARS-CoV-2 testing were common.

#### Genomic surveillance

During the pandemic, SARS-CoV-2 molecular laboratories in the Philippines could submit specimens (including those from inbound travelers) to their RESU to confirm eligibility for subsequent sequencing at RITM or PGC (Table 1). In April 2023, RITM’s turnaround time for lineage assignment was an average of two weeks from specimen receipt, and approximately 30 days from specimen collection, with an average of 47 specimens sequenced per week. From February 2020 through April 2023, RITM had submitted 2,866 SARS-CoV-2 genome sequences to the Global Initiative on Sharing All Influenza Data (GISAID) [21] with additional sequences uploaded by GECO-PH and other facilities. However, RITM’s molecular biology laboratory had only one pathogen-agnostic bioinformatician at the time of evaluation and in-country sequencing for influenza and RSV was not performed due to lack of laboratory space and personnel.

## Discussion

We summarized findings from the 2023 evaluation of COVID-19 and other respiratory virus surveillance systems in the Philippines. The country rapidly developed or adapted universal COVID-19 and sentinel surveillance methods and their respective information systems to meet large-scale COVID-19 pandemic response demands. The need for robust COVID-19 data to inform decision-making resulted in an increase in molecular testing and genomic sequencing capacity nationwide, including more than double the number of PhilHealth accredited SARS-CoV-2 testing laboratories in the first 2 years of the pandemic. At the time of the evaluation, throughput and timeliness for detecting SARS-CoV-2 exceeded that of any other respiratory virus. However, as resources available for viral respiratory surveillance declined in 2023, maintaining this expanded surveillance infrastructure was noted as a challenge. Rapid expansion led to fragmented data systems and confusion around stakeholder roles and responsibilities. Reporting completeness and timeliness varied across surveillance systems and time. Common challenges included process inefficiencies, data discrepancies, and heavier workloads on already limited staff.

Many of these phenomena are not unique to the Philippines. Countries worldwide created new COVID-19 surveillance systems, such as national case-based approaches in Italy [22], Morocco [23], and Croatia [24], a national outbreak surveillance system in Canada [25], and a screening information system in France [26]. Local adaptations to existing systems also occurred, such as expanding symptom-based traveler screening and quarantine programs [27], reconfiguring the District Health Information Software version 2 (DHIS2) for COVID-19 data needs [28] and leveraging SARI/ILI sentinel surveillance systems for COVID-19 surveillance [29]. Other adaptations included modifying Human Immunodeficiency Virus/Tuberculosis health information systems [30] and Acute Febrile Illness surveillance to support COVID-19 data collection [31]. Countries quickly increased COVID-19 testing through various strategies. South Korea established over 600 COVID-19 screening sites conducting approximately 20,000 tests daily by April 2020 [32]. Singapore rapidly implemented SARS-CoV-2 RT-PCR testing in all public hospitals [33], and India had 885 laboratories conducting over 125,000 COVID-19 tests daily by June 2020 [34]. The reactive pandemic response posture in many countries also led to similar challenges as observed in the Philippines. Initially, Spanish Autonomous Communes had incompatible data systems for COVID-19 surveillance [24], while Ghana and Uganda’s new digital systems were not interoperable with their existing DHIS2 system [35, 36], causing delays and data inconsistencies. In several European countries, varying governance and reporting channels across public health and healthcare facilities hampered early national surveillance data generation [24]. In Indonesia, information system challenges led to data discrepancies between national and sub-national levels and laboratory oversight by eleven government and private entities complicated standardization of operations [37]. Finally, persistence of multiple reporting methods in the U.S. by paper, phone, and fax, along with fragmented systems, created opportunities for mistakes and less timely reporting [38]. 

Recommendations from the evaluation were organized into two phases: (1) Right-sizing COVID-19 surveillance by scaling back universal case reporting, strengthening syndromic and sentinel surveillance networks, and integrating information systems and (2) planning for integration of influenza, SARS-CoV-2, and RSV into a single surveillance model. Despite declining case counts and increased population immunity through vaccination and/or infection in the Philippines, universal COVID-19 surveillance continued to operate in an acute emergency mode at the time of the evaluation. This is perhaps best demonstrated by COVID-19 case-based surveillance having the timeliest reporting from symptom onset to laboratory result of all evaluated surveillance systems, yet lower completeness of key variables than other systems. In emergency situations, lower data quality may be acceptable in favor of timeliness. However, data quality attributes like completeness become more important for sustainable routine surveillance and more achievable as universal surveillance is scaled back. Adapting universal COVID-19 surveillance to the current epidemiological situation by transitioning from daily to weekly or bi-weekly case reporting, reevaluating the utility of antigen testing for COVID-19 screening given widely accessible molecular testing, and focusing on COVID-19 hospitalizations and deaths could provide a better understanding of the severity burden. Additionally, downsizing contact tracing and traveler screening to targeted strategies while continuing regular sequence uploads to GISAID will support global SARS-CoV-2 variant tracking.

Efforts are underway to end universal COVID-19 surveillance and transition staff back to sentinel surveillance. This move could help maintain the commitment of the public health workforce and leadership, notable advancements in molecular testing and sequencing capacity, improved data reporting timeliness in some cases, and enhanced infrastructure for surveillance. Redirecting resources to strengthen the sentinel surveillance network will be important for integrated respiratory virus surveillance. Focusing staff time on sentinel surveillance would allow for monitoring of sentinel sites and routine auditing of data to ensure quality capture of key variables and resolution of discrepancies between reporting levels. Increased resources could also go towards providing standardized reporting tools and surveillance training for DSOs and clinicians. Similarly, initiating reporting of confirmed negative COVID-19 cases meeting ILI or SARI case definitions and encouraging SARI syndromic reporting from non-sentinel facilities can improve understanding of ILI/SARI trends as universal COVID-19 surveillance decreases. Additionally, assessing the quality and representativeness of existing sites by determining catchment areas to calculate rates can clarify whether regions are experiencing higher disease incidence or simply have more reporting sites, an important determination before adding sites. This conclusion aligns with an abstract from a 2018 assessment of the ILI surveillance system by the EB, which advised limiting it “to its current sites to ensure functionality while addressing system issues” [39]. Finally, assessing throughput capacity and detection quality of molecular laboratories testing for respiratory viruses can guide planning for multiplex molecular testing beyond sentinel sites.

Successful integrated viral respiratory disease surveillance will require harmonizing existing systems and procedures, integrating reporting tools (e.g. CIF, weekly or monthly reporting summaries, etc.) and testing algorithms, and prioritizing interoperable data systems to streamline reporting and reduce staff burden. Reviewing partnership agreements and defining stakeholder roles can ensure adequate resourcing and effective data sharing. Human resource sustainability remains a challenge due to heavy COVID-19 reporting requirements and loss of pandemic-era contracts. An inventory of surveillance workforce needs would support national human resource planning. Capacity building, developing best practices for ongoing training in public health surveillance, data management, and epidemiology, and establishing clear roles and responsibilities for surveillance staff can support robust reporting and staff retention. Although the Philippines, like many countries, may feel the impacts of decreased global funding post-pandemic, these recommendations are meant to reallocate and maximize resources as much as possible to sustain critical surveillance functions amidst challenging funding environments. These recommendations align with recent global strategies and resources from WHO that promote integrated respiratory virus surveillance. The recommendations to right-size universal COVID-19 surveillance, continue to monitor SARS-CoV-2 variants, and ensure robust sentinel and syndromic surveillance support the Mosaic Respiratory Surveillance Framework, which emphasizes multiple, fit-for-purpose approaches to provide a comprehensive picture of epidemic and pandemic respiratory viruses [40]. Similarly, the recommendation to integrate viral respiratory surveillance processes, tools, and systems aligns with WHO’s guidance for integrated sentinel surveillance for influenza, SARS-CoV-2, and RSV as part of reporting to eGISRS [6, 40]. Finally, recommendations for defining roles and cross-institutional communication, ensuring workforce sustainability, and maximizing laboratory capacity align with the concept of collaborative surveillance, which aims to strengthen “capacity and collaboration among diverse stakeholders, both within and beyond the health sector” to improve evidence for decision-making [8]. Further collaboration will benefit both respiratory and other infectious disease surveillance efforts.

### Limitations

There were several limitations to this evaluation. First, the evaluation design did not allow findings to be nationally representative; rather, it provided an in-depth view of surveillance operations at the national level and select sub-national sites in 2023, and operations have continued to evolve. Second, quantitative data collection at the sub-national level was challenging due to disjointed systems and staff’s incomplete view of the surveillance process, making comprehensive data pulls difficult. Third, assessing the quality of reporting and data capture for surveillance systems without routine performance monitoring was also challenging due in part to less oversight of these data.

## Conclusion

Insights from the Philippines’ COVID-19 and other respiratory virus surveillance evaluation offer a valuable case study as countries transition from COVID-19 emergency response to sustainable and collaborative respiratory virus surveillance. Rapid expansion of surveillance, data information systems, and molecular testing capacity during the pandemic led to siloed systems, unclear stakeholder roles and responsibilities, and difficulties with data reporting and management, challenges that are also documented in other countries. Despite these hurdles, advancements were made in molecular testing and genomic sequencing capacity. Enhanced surveillance infrastructure and the adaptability of the public health workforce demonstrated that timelier data reporting and specimen processing and establishment of RSV surveillance was possible, contributing to a more robust surveillance system for COVID-19 and other respiratory viruses. To maintain these benefits, surveillance of respiratory viruses can be adapted to the interpandemic period, which requires an integrated approach. While global guidance is critical to support post-pandemic surveillance enhancements and alignment to standards, country-specific experiences, like those highlighted in the Philippines’ evaluation, offer important insights and recommendations for strengthening viral respiratory surveillance post COVID-19 pandemic that may resonate with other countries in similar positions.

## Supplementary Information

Additional file 1. Health office, healthcare facility, and laboratory surveillance data structure, process, and flow information.

Additional file 2. COVID-19 and other respiratory virus surveillance key informant and focus group interview guide.

Additional file 3. Surveillance data collection at the national, regional and facility level to assess data quality, management, and reporting.

Additional file 4. Additional Table 1: List of evaluation sites visited by region — the Philippines, April 2023. Additional Table 2: Completeness of reporting for respiratory virus surveillance systems at national and sub-national levels during February 26 - April 1, 2023.

## Data Availability

Data is provided within the manuscript or supplementary information files. The datasets generated and/or analyzed during the current study are not publicly available as they are property of the Philippine Department of Health but are available from the corresponding author on reasonable request.

## Abbreviations

BOQ: Bureau of quarantine
CAR: Cordillera administrative region
CDRS: COVID-19 document repository system
CESU: City epidemiology surveillance unit
CIF: Case investigation form
CSQAU: COVID-19 surveillance and quick action unit
DOH: Department of health
DRU: Disease reporting unit
DSO: Disease surveillance officer
EB: Epidemiology bureau
EDCS-IS: Epidemic-prone disease case surveillance – information system
ESU: Epidemiology surveillance unit
GISAID: Global initiative on sharing all influenza data
GISRS: Global influenza surveillance and response system
HESU: Hospital epidemiology surveillance unit
IMCI: Integrated management of childhood illness
ILI: Influenza-like illness
LGU: Local government unit
LCP: Lung center of the Philippines
MIAA: Manila international airport authority
MESU: Municipal epidemiology surveillance unit
NCR: National capital region
NAIA: Ninoy Aquino international airport
ORV: Other respiratory virus
PESU: Provincial epidemiology surveillance unit
PGC: Philippine genome center
PHEIC: Public health emergency of international concern
PIDSR: Philippines integrated disease surveillance and response
PNIC: Philippine national influenza center
POE: Point of entry
QSIHSS: Quarantine services and international health surveillance system
RESU: Regional epidemiology surveillance unit
RHU: Rural health unit
RITM: Research institute for tropical medicine
RSV: Respiratory syncytial virus
RT-PCR: Reverse-transcript polymerase chain reaction
SARI: Severe acute respiratory infection
SCV-2: SARS-CoV-2
TKC: Tanod-Kontra COVID
WGS: Whole genome sequencing
WHO: World health organization
