# Supplementary material for: Optimizing surveillance post-pandemic: an evaluation of COVID-19 and other respiratory virus surveillance systems in the Philippines, April 2023
Source: BMC Public Health. 2025 Oct 8;25:3378. doi: 10.1186/s12889-025-24208-8 (PMC12506167; doi:10.1186/s12889-025-24208-8)
Supplement: Supplementary file 1 — Additional file 1. Health office, healthcare facility, and laboratory surveillance data structure, process, and flow information. [file 12889_2025_24208_MOESM1_ESM.docx]

**Data Structure, Process, and Flow Questions**

# **Form 1: Regional Office/Health Office Description Tool**

Name of Office Organization/Unit:

Type of Facility:

Name of Person Completing Form:

## Structure

1. For each of the following surveillance systems, about how many staff in this organization/unit spend all or part of their time working on or contributing to the system? (Place an ‘x’ on the line you wish to mark.)

COVID-19 Case Reporting & Testing 0 1-4 5+

Event-Based Surveillance 0 1-4 5+

Genomic sequencing/referrals 0 1-4 5+

RSV surveillance pilot 0 1-4 5+

Integrated (SCV-2, ILI/SARI) sentinel surveillance pilot 0 1-4 5+

Recorder Notes:

1. Does this organization/unit currently use any of the following tools for tracking individual case reports, testing/results, or contact tracing information? For each, indicate if the system is used only internally within this organization/unit, or if the system is used to share/transfer data to/from external parties.

Internal External

TKC ☐ ☐

CovidKaya ☐ ☐

CDRS ☐ ☐

PDSR ☐ ☐

Third Party Database System ☐ ☐

Google/Excel Spreadsheet ☐ ☐

Gmail/Email ☐ ☐

Recorder Notes:

## Data Process & Flow

1. From how many reporting sites does this organization/unit directly receive data submissions? (Place an ‘x’ on the line you wish to mark.)

Smaller/more local municipal public health offices/organizations 0 1-4 5+

Public/academic testing facilities 0 1-4 5+

Private testing facilities 0 1-4 5+

Hospitals 0 1-4 5+

Outpatient clinics/Providers 0 1-4 5+

Recorder Notes:

1. To which organizational levels/units does this organization/unit directly submit/report data?

Describe/Name:

- ☐ Larger/more national municipal public health offices/organizations
- ☐ Smaller/more local municipal public health offices/organizations
- ☐ Public dataset/dashboard
- ☐ Clinicians/Providers
- ☐ Patients

Recorder Notes:

1. In what form are data submitted from this organization/unit? Where are they submitted to?

Destination:

- ☐ Paper-Based
- ☐ Electronic
- ☐ Multiple/Other (describe):

Recorder Notes:

1. Describe the reliability of internet/network connectivity at this organization/unit.

- ☐Very reliable (sufficient bandwidth / infrequent outages)
- ☐ Somewhat reliable (some bandwidth limitations / occasional outages that delay reporting)
- ☐Very unreliable (major bandwidth limitations / frequent outages that delay reporting)
- ☐No connectivity

Recorder Notes:

1. Which of the following (respiratory infection) data-generation activities occur at this organization/unit (check all that apply)? For each activity, note which pathogen and/or surveillance system it occurs.

Pathogen/System

- ☐Creating paper-based case/lab reports
- ☐Summarizing paper-based reports (e.g., in a ledger)
- ☐Creating electronic case/lab reports
- ☐Entering data from paper-based reports into an electronic system
- ☐Other case/test data creation or entry (describe):

Recorder Notes:

1. Use the table below to describe the data files/types received at this organization/unit, and the data processing activities performed here. (Additional rows may be added as needed)

| **Received input data file name/description** | **Data Source** | **Format (e.g., paper, electronic)** | **Frequency**  **(e.g., weekly, monthly)** | **Processing activity (e.g., cleaning, merging)** | **Outputs from this file (e.g., merged file, upload)** |
| --- | --- | --- | --- | --- | --- |
| 1. |  |  |  |  |  |
| 2. |  |  |  |  |  |
| 3. |  |  |  |  |  |

Recorder Notes:

# **Form 2: Hospital/Health Clinic Description Tool**

Name of Facility/Organization/Unit:

Name of Person Completing Form:

## Structure

1. Does this facility use an electronic health record system for individual patient records?

- ☐Yes
- ☐No

Recorder Notes:

1. About how many staff at this facility in each of the following categories spend part or all of their time supporting patients with suspected respiratory infections? (Place an ‘x’ on the line you wish to mark.)

Physicians 0 1-4 5+

Nurses 0 1-4 5+

Clinical support staff 0 1-4 5+

Laboratory Staff 0 1-4 5+

Administrative staff 0 1-4 5+

Recorder Notes:

1. Which of the following best describes activities at this facility for patients presenting during overnight hours?

- ☐No overnight services
- ☐Emergency/triage only
- ☐Admission but limited workup/diagnosis/testing
- ☐Full services performed

Recorder Notes:

1. Describe/draw the process or algorithm used to assess whether a respiratory specimen is taken for diagnostic testing from a patient presenting with a respiratory infection or symptoms of acute respiratory illness.
2. Do any of the following factors limit the number of respiratory specimens that can be collected?

- ☐Insufficient number of daytime staff
- ☐Insufficient number of overnight staff
- ☐Staff trained in specimen collection
- ☐Specimen collection supplies
- ☐Refrigerator/freezer space
- ☐Expected lab testing capacity/turnaround time
- ☐Institutional costs (that the facility/health system pay)
- ☐Out-of-pocket costs (that the patient pays)
- ☐Other (describe below):

Recorder Notes:

1. What laboratory/laboratories perform microbiological testing of respiratory specimens collected at this facility?

1. With regards to the storage and transportation of respiratory specimens for microbiological testing, which of the following occurs at this facility (check all that apply)?

- ☐Immediately refrigerated on-site
- ☐Immediately frozen on-site
- ☐Stored in cooler/on ice on-site
- ☐Kept at room temperature (no cooling before pickup)
- ☐Transported by health facility staff to laboratory
- ☐Picked up by laboratory staff
- ☐Transported by private courier company/service

Recorder Notes:

## Data Process & Flow

1. If patients are diagnosed with respiratory infections without microbiological diagnostic tests, are these diagnoses recorded in any of the following ways?

- ☐Entry in CDRS, CovidKaya, TKC, or other DOH database
- ☐Entry in non-electronic Patient Record
- ☐Entry in electronic patient record
- ☐Entry in electronic clinic/hospital surveillance database
- ☐Other (describe):

Recorder Notes:

1. Which of the following best describes how laboratory-confirmed diagnoses are reported to public health organizations.

- ☐Submission of paper case report form
- ☐Submission of electronic case report
- ☐Submission of paper aggregate report
- ☐Submission of electronic aggregate report/data file

Recorder Notes:

1. Are syndromic diagnoses (made without microbiological test results) made for respiratory infections/illness (e.g. COVID-19, influenza, ARI)?

- ☐Yes
- ☐No

Recorder Notes:

1. Which of the following best describes how syndromic diagnoses (those made without microbiological test results) are reported to public health organizations.

- ☐Submission of paper case report form
- ☐Submission of electronic case report
- ☐Submission of paper aggregate report
- ☐Submission of electronic aggregate report/data file
- ☐Syndromic diagnoses not reported
- ☐N/A (no syndromic diagnoses made here)

Recorder Notes:

# **Form 3: Laboratory Description Tool**

Name of Laboratory/Organization/Unit:

Type of Facility:

Name of Person Completing Form:

## Structure

1. How many staff perform testing of respiratory pathogens at this facility either some of the time or all of the time? (Place an ‘x’ on the line you wish to mark.)

- ☐ 0
- ☐1-4
- ☐5+

Recorder Notes:

1. How many staff perform each of the following functions, either some of the time or all the time, in a typical day? (Place an ‘x’ on the line you wish to mark.)

Specimen receipt/accessioning 0 1-4 5+

Performing diagnostic tests/assays 0 1-4 5+

Recording results 0 1-4 5+

Quality control 0 1-4 5+

Recorder Notes:

1. Which of the following best describes activities taken at this facility during overnight hours?

- ☐No overnight services
- ☐Accessioning but no testing
- ☐Accessioning and limited respiratory virus testing
- ☐Full overnight services

Recorder Notes:

1. Which of the following assays does this lab perform?

- ☐ SARS-CoV-2 NAAT (e.g., RT-PCR)
- ☐ SARS-CoV-2 + Influenza Combined Test
- ☐ SARS-CoV-2 Viral Load Quantification
- ☐ SARS-CoV-2 Serology
- ☐ SARS-CoV-2 Sequencing - no specimens sent for sequencing
- ☐ SARS-CoV-2 Culture
- ☐ Other SARS-CoV-2 Assay – describe:
- ☐ Influenza A/B NAAT
- ☐ RSV NAAT
- ☐ HAdV NAAT
- ☐ HAdV Typing
- ☐ Multiplex/multi-pathogen testing – describe:

Recorder Notes:

1. Which of the following occurs for the storage and transportation of respiratory specimens for microbiological testing at this facility (check all that apply)?

- ☐ At room temperature before arrival
- ☐ Refrigerated/on-ice before arrival
- ☐ Frozen/on dry ice before arrival
- ☐ Transported by health facility staff to laboratory
- ☐ Transported up by laboratory staff
- ☐ Transported by private courier company/service
- ☐ At room temperature after accessioning
- ☐ Refrigerated after accessioning
- ☐ Frozen after accessioning

Recorder Notes:

1. Are samples/aliquots retained after diagnostic testing is complete?

- ☐ Yes
- ☐ No

Recorder Notes:

1. Does this organization/unit currently use any of the following tools for tracking individual case reports, testing/results, or contact tracing information? For each, indicate if the system is used only internally within this organization/unit, or if the system is used to share/transfer data to/from external parties.

Internal External

TKC ☐ ☐

CovidKaya ☐ ☐

CDRS ☐ ☐

PDSR ☐ ☐

Third Party Database System ☐ ☐

Google/Excel Spreadsheet ☐ ☐

Gmail/Email ☐ ☐

Recorder Notes:

## Data Process & Flow

1. Which of the following identifiers are used for each specimen (check all that apply):

- ☐ Patient Name
- ☐ DOB
- ☐ Patient address/phone/ID no.
- ☐ Date of collection
- ☐ Specimen UID
- ☐ Accompanying form with identifiers

Recorder Notes:

1. Which of the following methods are used to report test results to providers/patients?

- ☐ Electronic health record system
- ☐ Patient portal system
- ☐ Phone call
- ☐ Text message/SMS
- ☐ Email
- ☐ Other (describe):

Recorder Notes:

1. Which of the following best describes how respiratory virus test results are reported to public health organizations.

- ☐ Submission of paper case report form
- ☐ Submission of electronic case report
- ☐ Submission of paper aggregate report
- ☐ Submission of electronic aggregate report/data file

Recorder Notes:
