# Supplementary material for: Optimizing surveillance post-pandemic: an evaluation of COVID-19 and other respiratory virus surveillance systems in the Philippines, April 2023
Source: BMC Public Health. 2025 Oct 8;25:3378. doi: 10.1186/s12889-025-24208-8 (PMC12506167; doi:10.1186/s12889-025-24208-8)
Supplement: Supplementary file 2 — Additional file 2. COVID-19 and other respiratory virus surveillance key informant and focus group interview guide. [file 12889_2025_24208_MOESM2_ESM.docx]

**COVID-19 Surveillance Evaluation in the Philippines**Key Informant and Focus Group Discussion Questions

**Introduction to Activity**

Thank you for taking the time to speak with us today. The purpose of this discussion is to better understand **COVID-19 and other respiratory virus surveillance systems** in the Philippines from the perspectives of those who use them the most: e.g., epidemiologists, DSOs, data managers, decision-makers, etc. We are particularly interested in your thoughts and experiences in operating these systems, how useful you find them and the data they generate, any challenges you experience, and recommendations you may have for improvement.

We understand that COVID-19 and other respiratory disease surveillance is likely just ONE of your many job responsibilities, however, we will focus this discussion on these surveillance systems ONLY for the purposes of our visit.

While we are requesting to take down your name for this interview, all responses across participants and sites will be aggregated, and no individual response will be linked to any participant’s name once included in the data analysis.

Therefore, we appreciate your honest thoughts and insights and into the following questions. Any questions before we start?

Thank you!

***For groups of more than 2 individuals, please take general notes on the staff's background***

| 1. **Site name and type (e.g. hospital)** |  |
| --- | --- |
| 1. **Date of interview** |  |
| 1. **Name/organization of interviewer** |  |
| 1. **Name of interviewee** |  |
| 1. **Job title/responsibilities of the interviewee** | Interviewee #1: |
|  | Interviewee #2: |
|  | Interviewee #3: |
| 1. **Length of time interviewee has been in current position** | Interviewee #1: |
|  | Interviewee #2: |
|  | Interviewee #3: |
| 1. **The surveillance system(s) the interviewee is primarily involved in** | Interviewee #1: |
|  | Interviewee #2: |
|  | Interviewee #3: |

**Part 1: Questions for All Staff**

***Training***

1. Can you start by describing your job and responsibilities? With respect to **respiratory disease surveillance**, what activities are you responsible for during a typical day?

| Interviewee #1: |  |
| --- | --- |
| Interviewee #2: |  |
| Interviewee #3: |  |

1. What training did you receive *(insert time period if desired, e.g., the last 2 years)* to perform these **respiratory disease** **surveillance** activities?

- *Probe: Have you received any refresher or on-the-job training? When was that? What did the training entail? How did or didn’t this help you conduct your current job responsibilities?*

| Interviewee #1: |  |
| --- | --- |
| Interviewee #2: |  |
| Interviewee #3: |  |

1. What training do you feel you are missing in order to best perform your responsibilities regarding **respiratory disease surveillance?**

| Interviewee #1: |  |
| --- | --- |
| Interviewee #2: |  |
| Interviewee #3: |  |

***System and Data Usefulness***

There are multiple surveillance systems in the country that collect data on **respiratory diseases** *(the facilitator lists a few)*.

1. In your opinion, are there differences between the systems in terms of which is the easiest to use? To maintain? *(Consider information systems, reporting requirements, data management, etc.)*

| Interviewee #1: |  |
| --- | --- |
| Interviewee #2: |  |
| Interviewee #3: |  |

1. Thinking of these same **respiratory disease surveillance** systems, can you describe how the data from each system is used for decision-making? Are the systems used by different stakeholders or for different purposes?

- *Probe: Who do you see as key users of the surveillance data? At your facility? In your city or region? At the national level?*
- *Probe: How do they use the data? Are there any decisions that are informed by the current surveillance data? If so, what?*
- *Probe: If needed, probe by surveillance system.*

| Interviewee #1: |  |
| --- | --- |
| Interviewee #2: |  |
| Interviewee #3: |  |

***Key Challenges***

1. What challenges do you currently encounter in your day-to-day **respiratory disease surveillance** responsibilities?

*(Allow participant(s) to answer first. Consider prompting on the areas below if the participant does not mention them.)*

| **Challenge Areas** | **Response** |
| --- | --- |
| Tasks   - Case identification/reporting? - Testing? - Analysis, dissemination (e.g. bulletins), and use of data? | Interviewee #1: |
|  | Interviewee #2: |
|  | Interviewee #3: |
| Human Resources | Interviewee #1: |
|  | Interviewee #2: |
|  | Interviewee #3: |
| Funding | Interviewee #1: |
|  | Interviewee #2: |
|  | Interviewee #3: |
| Others | Interviewee #1: |
|  | Interviewee #2: |
|  | Interviewee #3: |

1. In your opinion, how could these challenges be addressed?

| Interviewee #1: |  |
| --- | --- |
| Interviewee #2: |  |
| Interviewee #3: |  |

***Recommendations***

1. Thinking more broadly about the systems as a whole, what other recommendations do you have to improve surveillance for **respiratory diseases including COVID-19 in the Philippines?**
   - *Probe: Think about processes, documentation, and training, that were received for (X surveillance system, or Y surveillance system).*
   - *Probe: Surveillance for respiratory diseases changed a lot during the pandemic. Which of these changes should be maintained? Which should be maintained long term? Are there additional changes that should be considered?*
   - *Probe: Are there activities that are duplicative across surveillance systems (e.g., the same information collected multiple times)?*

| Interviewee #1: |  |
| --- | --- |
| Interviewee #2: |  |
| Interviewee #3: |  |

***The next section includes role-specific questions. Please advance to the appropriate category for this evaluation site.***

**Part 2: Central Level Surveillance**

***System and Data Usefulness***

1. Is there any additional COVID-19 or other respiratory disease surveillance data that would be helpful for decision-making that are ***not*** currently collected?
   1. If yes, please explain.

| Interviewee #1: |  |
| --- | --- |
| Interviewee #2: |  |
| Interviewee #3: |  |

1. Are there routine COVID-19 or other respiratory disease surveillance data you currently receive that are ***not used***?
   1. If yes, please explain (e.g., it’s duplicative, incomplete, irrelevant, etc.)

| Interviewee #1: |  |
| --- | --- |
| Interviewee #2: |  |
| Interviewee #3: |  |

1. Are there processes of supportive supervision in place currently to ensure the quality of COVID-19 and other respiratory disease surveillance systems? If so, please describe them.

| **Questions** | **Response** |
| --- | --- |
| Who conducts the supervision? | Interviewee #1: |
|  | Interviewee #2: |
|  | Interviewee #3: |
| How often? | Interviewee #1: |
|  | Interviewee #2: |
|  | Interviewee #3: |
| What does it entail? Are there SOPs to structure this process? | Interviewee #1: |
|  | Interviewee #2: |
|  | Interviewee #3: |
| Is supervision done separately for each surveillance system or are supervision visits to the same region/facility coordinated across departments? | Interviewee #1: |
|  | Interviewee #2: |
|  | Interviewee #3: |
| Is this process the primary mechanism to provide feedback to staff? If not, please explain. | Interviewee #1: |
|  | Interviewee #2: |
|  | Interviewee #3: |

1. Is there anything else you’d like to add that we haven’t discussed, or anything else you feel we should know for our evaluation of COVID-19 and other respiratory disease surveillance systems in the Philippines?

| Interviewee #1: |
| --- |
| Interviewee #2: |
| Interviewee #3: |

**Part 3: Intermediate Level Surveillance / Data Manager**

***System and Data Usefulness***

1. How is the performance of reporting sites evaluated (e.g., identifying those who are not routinely reporting or are submitting incomplete reports)?

- *Probe: Who is responsible for reviewing performance?*
- *Probe: What indicators/processes are used to evaluate performance?*

| Interviewee #1: |
| --- |
| Interviewee #2: |
| Interviewee #3: |

1. Please describe any processes for following up with facilities that are identified as underperforming.
   1. Who performs this follow-up?
   2. In your opinion, how well is this process currently working?

| Interviewee #1: |
| --- |
| Interviewee #2: |
| Interviewee #3: |

1. Please describe the process for checking unusual or unexpected respiratory disease surveillance data.
   1. What do you do if you find anything unusual or unexpected?

| Interviewee #1: |
| --- |
| Interviewee #2: |
| Interviewee #3: |

1. Is there anything else you’d like to add that we haven’t discussed, or anything else you feel we should know for our evaluation of COVID-19 and other respiratory disease surveillance systems in the Philippines?

| Interviewee #1: |
| --- |
| Interviewee #2: |
| Interviewee #3: |

**Part 4: Disease Reporting Unit Staff**

***System and Data Usefulness***

1. Please describe the patient population that you serve at this facility (consider geographic and socioeconomic characteristics, age, ethnic groups, etc.)
2. Are there multiple respiratory disease surveillance systems operating in this facility? If so, can you describe how they operate independently or overlap in any way (e.g. data collection processes, staff involved, other) within this facility?

- *Probe: Are the same staff involved in data collection and lab activities for cases eligible for each system or are there distinct staff? Why? How is that working?*
- *Probe: Can you describe differences in eligibility comparing these respiratory disease surveillance systems?*
- *Probe: Can you describe differences in SOPs comparing these respiratory disease surveillance systems?*
- *Probe: Consider case reports and lab confirmation, access to vaccination data, etc.*

| Interviewee #1: |
| --- |
| Interviewee #2: |
| Interviewee #3: |

1. Are there standard targets for timely respiratory disease data collection and reporting in your facility?
   1. If yes, what are they? How often does this facility achieve those timeliness targets?
      1. If timeliness targets are not frequently met, what are the specific barriers to timely data collection and reporting?

| Interviewee #1: |
| --- |
| Interviewee #2: |
| Interviewee #3: |

1. Please describe the most recent supervision visit you received.

*(Note: countries may be unfamiliar with the term “supervision visit.” If necessary, please explain that it refers to upper administrative surveillance levels conducting site visits to ensure optimal staff performance according to standards/protocols and troubleshooting any issues encountered.)*

| **Questions:** | **Response** |
| --- | --- |
| When did it occur? | Interviewee #1: |
|  | Interviewee #2: |
|  | Interviewee #3: |
| Who performed it? | Interviewee #1: |
|  | Interviewee #2: |
|  | Interviewee #3: |
| What was the purpose of the visit and what did it entail? | Interviewee #1: |
|  | Interviewee #2: |
|  | Interviewee #3: |

1. Is there anything else you’d like to add that we haven’t discussed, or anything else you feel we should know for our evaluation of COVID-19 and other respiratory disease surveillance systems in the Philippines?

| Interviewee #1: |
| --- |
| Interviewee #2: |
| Interviewee #3: |

**Thank you so much for taking the time to speak with us today!**
