# Supplementary material for: Optimizing surveillance post-pandemic: an evaluation of COVID-19 and other respiratory virus surveillance systems in the Philippines, April 2023
Source: BMC Public Health. 2025 Oct 8;25:3378. doi: 10.1186/s12889-025-24208-8 (PMC12506167; doi:10.1186/s12889-025-24208-8)
Supplement: Supplementary file 3 — Additional file 3. Surveillance data collection at the national, regional and facility level to assess data quality, management, and reporting. [file 12889_2025_24208_MOESM3_ESM.docx]

| **Purpose:** | To capture data at the national, regional and facility level to assess data quality, management, and reporting (i.e. looking at reporting, timeliness, completeness and representativeness metrics). | | | | | | | |  |  |  |  |  |
| --- | --- | --- | --- | --- | --- | --- | --- | --- | --- | --- | --- | --- | --- |
| **Surveillance Systems Evaluating:** | COVID-19 Case Surveillance (COVIDKaya/TKC) | | | |  |  |  |  |  |  |  |  |  |
|  | COVID-19 Genomic Sequencing | | |  |  |  |  |  |  |  |  |  |  |
|  | Traveler Screening (focus on COVID-19) | | |  |  |  |  |  |  |  |  |  |  |
|  | DOH Data Collect (hospital census) | | |  |  |  |  |  |  |  |  |  |  |
|  | ILI / SARI Surveillance (PIDSR) | | |  |  |  |  |  |  |  |  |  |  |
|  | ILI / SARI Sentinel Surveillance (PIDSR) | | |  |  |  |  |  |  |  |  |  |  |
|  | RSV Surveillance Pilot | |  |  |  |  |  |  |  |  |  |  |  |
|  |  |  |  |  |  |  |  |  |  |  |  |  |  |
| **General Notes/Instructions:** | 1. If evaluating more than one surveillance system at a site, copy/paste the table within a tab to collect data for each surveillance system | | | | | | | | | |  |  |  |
|  | 2. The same 25 samples/observations can be used to complete the tables across defined epi weeks 9-13 for each table where this is noted | | | | | | | | | |  |  |  |
|  | 3. When capturing dates, epi week corresponds to date of specimen collection | | | | | | |  |  |  |  |  |  |
|  | Epi weeks 9-13 correspond to the following calendar weeks in the year 2023 below: | | | | | | |  |  |  |  |  |  |
|  | Epi week 9 - Week of Feb 26 | | |  |  |  |  |  |  |  |  |  |  |
|  | Epi week 10 - Week of Mar 5 | | |  |  |  |  |  |  |  |  |  |  |
|  | Epi week 11 - Week of Mar 12 | | |  |  |  |  |  |  |  |  |  |  |
|  | Epi Week 12 - Week of Mar 19 | | |  |  |  |  |  |  |  |  |  |  |
|  | Epi Week 13 - Week of Mar 26 | | |  |  |  |  |  |  |  |  |  |  |
|  |  |  |  |  |  |  |  |  |  |  |  |  |  |
| **Section/table specific Instructions:** | Complete all tables for all surveillance systems being evaluated unless otherwise indicated | | | | | | |  |  |  |  |  |  |
|  | **National Level** | **Regional/Local Level** | **Hospital/Facility Level** |  |  |  |  |  |  |  |  |  |  |
|  | 1.1 | 1.1 |  |  |  |  |  |  |  |  |  |  |  |
|  | 1.2 | 1.2 | 1.2 |  |  |  |  |  |  |  |  |  |  |
|  | 1.3 | 1.3 | 1.3 |  |  |  |  |  |  |  |  |  |  |
|  | 2.1 | 2.1 | 2.1 |  |  |  |  |  |  |  |  |  |  |
|  | 4.1* |  |  |  |  |  |  |  |  |  |  |  |  |
|  | 4.2* |  |  |  |  |  |  |  |  |  |  |  |  |
|  | *Tables 4.1 and 4.2 are to be completed at the National level only, with confirmation of data at the regional/local level for both | | | | | | | | |  |  |  |  |
|  |  |  |  |  |  |  |  |  |  |  |  |  |  |
| **Data Collection Level** | **Contents** | List of tables for data abstraction below | | |  |  |  |  |  |  |  |  |  |
|  | **Section 1:** | Data Quality - Reporting completeness/timeliness for each surveillance system | | | | | |  |  |  |  |  |  |
| National and Regional/Local level | **Table 1.1** | Completeness: Capturing information on reporting sites to assess completeness of reporting | | | | | | |  |  |  |  |  |
| National, Regional/Local, and Hospital/Facility level | **Table 1.2** | Completeness – Review key variables from the reporting forms (or CIF) for completeness | | | | | | |  |  |  |  |  |
| National, Regional/Local and Hospital/Facility Level | **Table 1.3** | Data Quality/management: assessing the number of cases without confirmed lab test by surveillance system | | | | | | | |  |  |  |  |
|  |  |  |  |  |  |  |  |  |  |  |  |  |  |
|  | **Section 2:** | Assessing timeliness (speed) between steps in all public health surveillance systems from notification to verification and results reporting (for laboratory-confirmed cases) | | | | | | | | | | | |
| National, Regional/Local, Hospital/Facility level | **Table 2.1** | Timeliness: Capturing dates of symptom onset, collection, receipt, testing and reporting to assess time between steps | | | | | | | | |  |  |  |
|  |  |  |  |  |  |  |  |  |  |  |  |  |  |
|  | **Section 3:** | Representativeness – assessing surveillance system ability to describe occurrence of health events over time and the distribution by person, place and time | | | | | | | | | | |  |
| National level only* | **Table 3.1** | Map reporting facilities/catchment areas for each surveillance system | | | | | |  |  |  |  |  |  |
| National level only* | **Table 3.2** | Epidemic curve by data source: weekly case counts by age groups. Note: If only paper records available, complete epi weeks for 2023 only | | | | | | | | | |  |  |
| *confirmation of data at some regional/local levels | |  |  |  |  |  |  |  |  |  |  |  |  |

|  | ***National and Regional/provincial Level*** | | |  |  |  |
| --- | --- | --- | --- | --- | --- | --- |
| **1.1: Completeness: Capturing information on reporting sites to assess completeness of reporting.** | | | | | |  |
| ***Instructions:*** |  |  |  |  |  |  |
| 1. Review epi weeks 9-13 (February 26-April 1, 2023) for each surveillance system (adjust reporting period as needed). | | | | | | |
| 2. Complete table for each surveillance system being evaluated | | |  |  |  |  |
| 3. Zero reporting from any site should be included | | |  |  |  |  |
| **Name of Surveillance System:** | |  |  |  |  |  |
| **Epi week** | **No. of sites reporting during weekly reporting period** (on time) | **Total No. of sites** | **Percent of sites reporting** |  |  |  |
| 9 (Feb 26-Mar 4, 2023) |  |  | #DIV/0! |  |  |  |
| 10 (Mar 5-11, 2023) |  |  | #DIV/0! |  |  |  |
| 11 (Mar 12-18, 2023) |  |  | #DIV/0! |  |  |  |
| 12 (Mar 19-25, 2023) |  |  | #DIV/0! |  |  |  |
| 13 (Mar 26-Apr 1, 2023) |  |  | #DIV/0! |  |  |  |
| Average (total/5) | 0 | 0 | #DIV/0! |  |  |  |

|  | ***National, Regional/Local, and Hospital/Facility level*** | | | | | | | | | | |  |
| --- | --- | --- | --- | --- | --- | --- | --- | --- | --- | --- | --- | --- |
| **1.2: Completeness – Review key fields from the reporting forms (or CIF) for completeness** | | | | | | | |  |  |  |  |  |
|  |  |  |  |  |  |  |  |  |  |  |  |  |
| ***Instructions:*** |  |  |  |  |  |  |  |  |  |  |  |  |
| 1. Review epi weeks 9-13 (February 26-April 1, 2023) in national, regional or facility data; collect missing/unknown values for key variables as follows: | | | | | | | | | | | | |
| *n = number of observations with blank or unknown value, N=Total number of observations for epi week* | | | | | | | | | |  |  |  |
| 2. Complete table for each surveillance system being evaluated | | | | |  |  |  |  |  |  |  |  |
| 3. IF a key variable listed is not captured for this surveillance system, highlight row in gray and make a note in the row of that variable | | | | | | | | | | | |  |
| **Name of Surveillance System:** |  |  |  |  |  |  |  |  |  |  |  |  |
|  | **Epi Week 9** | | **Epi week 10** | | **Epi week 11** | | **Epi week 12** | | **Epi week 13** | |  |  |
|  | Feb 26-Mar 4, 2023 | | Mar 5-11, 2023 | | Mar 12-18, 2023 | | Mar 19-25, 2023 | | Mar 26-Apr 1, 2023 | |  |  |
| **Question/Variable** | **n** | **N** | **n** | **N** | **n** | **N** | **n** | **N** | **n** | **N** |  |  |
| Age |  |  |  |  |  |  |  |  |  |  |  |  |
| Sex |  |  |  |  |  |  |  |  |  |  |  |  |
| Residence |  |  |  |  |  |  |  |  |  |  |  |  |
| Vaccination status  (completed or not) |  |  |  |  |  |  |  |  |  |  |  |  |
| Illness onset date |  |  |  |  |  |  |  |  |  |  |  |  |
| Specimen collection date  (if a confirmed case) |  |  |  |  |  |  |  |  |  |  |  |  |
| Hospital admission date  (if hospitalized) |  |  |  |  |  |  |  |  |  |  |  |  |
| Hospital discharge date  (if hospitalized) |  |  |  |  |  |  |  |  |  |  |  |  |
| Signs/Symptoms |  |  |  |  |  |  |  |  |  |  |  |  |
| Lab test results  (for confirmed cases) |  |  |  |  |  |  |  |  |  |  |  |  |
| Outcome (survived or not) |  |  |  |  |  |  |  |  |  |  |  |  |

| ***National, Regional/Local, and Hospital/Facility level***  **2: Data Quality/management: assessing the number of cases without confirmed lab test by surveillance system**  Record the information below for epi weeks 9-13 for each surveillance system | | | | | | |
| --- | --- | --- | --- | --- | --- | --- |
| **Surveillance System** | **Epi Weeks 9-13** | **Number of cases with POSITIVE result** | **Number of cases with NEGATIVE result** | **Number of cases that testing was not performed** | **Number of cases tested** | **Number of total cases reported** |
| COVID Case reporting | 9 (Feb 26-Mar 4, 2023) |  |  |  |  |  |
|  | 10 (Mar 5-11, 2023) |  |  |  |  |  |
|  | 11 (Mar 12-18, 2023) |  |  |  |  |  |
|  | 12 (Mar 19-25, 2023) |  |  |  |  |  |
|  | 13 (Mar 26-Apr 1, 2023) |  |  |  |  |  |
| Genomic sequencing/referrals | 9 (Feb 26-Mar 4, 2023) |  |  |  |  |  |
|  | 10 (Mar 5-11, 2023) |  |  |  |  |  |
|  | 11 (Mar 12-18, 2023) |  |  |  |  |  |
|  | 12 (Mar 19-25, 2023) |  |  |  |  |  |
|  | 13 (Mar 26-Apr 1, 2023) |  |  |  |  |  |
| Traveler Screening | 9 (Feb 26-Mar 4, 2023) |  |  |  |  |  |
|  | 10 (Mar 5-11, 2023) |  |  |  |  |  |
|  | 11 (Mar 12-18, 2023) |  |  |  |  |  |
|  | 12 (Mar 19-25, 2023) |  |  |  |  |  |
|  | 13 (Mar 26-Apr 1, 2023) |  |  |  |  |  |
| RSV surveillance pilot | 9 (Feb 26-Mar 4, 2023) |  |  |  |  |  |
|  | 10 (Mar 5-11, 2023) |  |  |  |  |  |
|  | 11 (Mar 12-18, 2023) |  |  |  |  |  |
|  | 12 (Mar 19-25, 2023) |  |  |  |  |  |
|  | 13 (Mar 26-Apr 1, 2023) |  |  |  |  |  |
| ILI Surveillance | 9 (Feb 26-Mar 4, 2023) |  |  |  |  |  |
|  | 10 (Mar 5-11, 2023) |  |  |  |  |  |
|  | 11 (Mar 12-18, 2023) |  |  |  |  |  |
|  | 12 (Mar 19-25, 2023) |  |  |  |  |  |
|  | 13 (Mar 26-Apr 1, 2023) |  |  |  |  |  |
| ILI Sentinel Surveillance | 9 (Feb 26-Mar 4, 2023) |  |  |  |  |  |
|  | 10 (Mar 5-11, 2023) |  |  |  |  |  |
|  | 11 (Mar 12-18, 2023) |  |  |  |  |  |
|  | 12 (Mar 19-25, 2023) |  |  |  |  |  |
|  | 13 (Mar 26-Apr 1, 2023) |  |  |  |  |  |
| SARI Surveillance | 9 (Feb 26-Mar 4, 2023) |  |  |  |  |  |
|  | 10 (Mar 5-11, 2023) |  |  |  |  |  |
|  | 11 (Mar 12-18, 2023) |  |  |  |  |  |
|  | 12 (Mar 19-25, 2023) |  |  |  |  |  |
|  | 13 (Mar 26-Apr 1, 2023) |  |  |  |  |  |
| SARI Sentinel Surveillance | 9 (Feb 26-Mar 4, 2023) |  |  |  |  |  |
|  | 10 (Mar 5-11, 2023) |  |  |  |  |  |
|  | 11 (Mar 12-18, 2023) |  |  |  |  |  |
|  | 12 (Mar 19-25, 2023) |  |  |  |  |  |
|  | 13 (Mar 26-Apr 1, 2023) |  |  |  |  |  |

| **3: Timeliness – Capturing dates of symptom onset, collection, receipt, testing and reporting to assess time between steps** | | | | | | | | | | | | | |
| --- | --- | --- | --- | --- | --- | --- | --- | --- | --- | --- | --- | --- | --- |
| Record the information below for 5 randomly selected samples from epi weeks 9-13 (February 26-April 1, 2023) for each surveillance system.  If evaluating multiple surveillance systems, repeat the table for each system | | | | | | | | | | | |  |  |
|  |  |  |  |  |  |  |  |  |  |  |  |  |  |
| **Name of Surveillance System:** | |  |  |  |  |  |  |  |  |  |  |  |  |
| **Observation  (replace with unique caseID)** | **Epi Week** | **Samples for _________ (disease test name)** | **Date of Symptom Onset** **(dd/mm/yy)** *(if date not available, collect earliest date identified and note in comments)* | **Date of diagnosis/first consultation (dd/mm/yy)** | **Date of specimen collection (dd/mm/yy)** | **Date received by laboratory (dd/mm/yy)** | **Date test performed (dd/mm/yy)** | **Date result reported back to collection site** **(dd/mm/yy)** | **Date of report to surveillance unit (dd/mm/yy)** | **Type of test (antigen/RT-PCR)** if not a confirmed case, leave blank and add a note | **Result of Test (positive/negative)** | **Notes** | **Reporting difference (in days), D-C** |
|  | 9 (Feb 26-Mar 4, 2023) |  |  |  |  |  |  |  |  |  |  |  |  |
|  | 9 (Feb 26-Mar 4, 2023) |  |  |  |  |  |  |  |  |  |  |  |  |
|  | 9 (Feb 26-Mar 4, 2023) |  |  |  |  |  |  |  |  |  |  |  |  |
|  | 9 (Feb 26-Mar 4, 2023) |  |  |  |  |  |  |  |  |  |  |  |  |
|  | 9 (Feb 26-Mar 4, 2023) |  |  |  |  |  |  |  |  |  |  |  |  |
|  | 10 (Mar 5-11, 2023) |  |  |  |  |  |  |  |  |  |  |  |  |
|  | 10 (Mar 5-11, 2023) |  |  |  |  |  |  |  |  |  |  |  |  |
|  | 10 (Mar 5-11, 2023) |  |  |  |  |  |  |  |  |  |  |  |  |
|  | 10 (Mar 5-11, 2023) |  |  |  |  |  |  |  |  |  |  |  |  |
|  | 10 (Mar 5-11, 2023) |  |  |  |  |  |  |  |  |  |  |  |  |
|  | 11 (Mar 12-18, 2023) |  |  |  |  |  |  |  |  |  |  |  |  |
|  | 11 (Mar 12-18, 2023) |  |  |  |  |  |  |  |  |  |  |  |  |
|  | 11 (Mar 12-18, 2023) |  |  |  |  |  |  |  |  |  |  |  |  |
|  | 11 (Mar 12-18, 2023) |  |  |  |  |  |  |  |  |  |  |  |  |
|  | 11 (Mar 12-18, 2023) |  |  |  |  |  |  |  |  |  |  |  |  |
|  | 12 (Mar 19-25, 2023) |  |  |  |  |  |  |  |  |  |  |  |  |
|  | 12 (Mar 19-25, 2023) |  |  |  |  |  |  |  |  |  |  |  |  |
|  | 12 (Mar 19-25, 2023) |  |  |  |  |  |  |  |  |  |  |  |  |
|  | 12 (Mar 19-25, 2023) |  |  |  |  |  |  |  |  |  |  |  |  |
|  | 12 (Mar 19-25, 2023) |  |  |  |  |  |  |  |  |  |  |  |  |
|  | 13 (Mar 26-Apr 1, 2023) |  |  |  |  |  |  |  |  |  |  |  |  |
|  | 13 (Mar 26-Apr 1, 2023) |  |  |  |  |  |  |  |  |  |  |  |  |
|  | 13 (Mar 26-Apr 1, 2023) |  |  |  |  |  |  |  |  |  |  |  |  |
|  | 13 (Mar 26-Apr 1, 2023) |  |  |  |  |  |  |  |  |  |  |  |  |
|  | 13 (Mar 26-Apr 1, 2023) |  |  |  |  |  |  |  |  |  |  |  |  |

|  | ***NATIONAL LEVEL ONLY -- CONFIRM REGIONAL/PROVINCIAL DATA WITH RESU/CESU/PESUs*** | | |
| --- | --- | --- | --- |
| **3.1: Map reporting facilities/catchment areas for each surveillance system** | | |  |
| ***Instructions:*** |  |  |  |
| 1. Repeat table for each surveillance system being evaluated | | |  |
| 2. Report zero cases if no cases reported for a particular province/region during indicated time period | | | |

| **Name of Surveillance System:** | |  |  |  |
| --- | --- | --- | --- | --- |
| **Total confirmed cases (Jan 2021 - Mar 2023)** | **Total Population** | **Region** | **Province** | **City Designation** |
|  |  | **---** | --- |  |
|  |  | **Region I (Ilocos)** | --- |  |
|  |  | **Region I (Ilocos)** | DAGUPAN CITY | ICC |
|  |  | **Region I (Ilocos)** | ILOCOS NORTE |  |
|  |  | **Region I (Ilocos)** | ILOCOS SUR |  |
|  |  | **Region I (Ilocos)** | LA UNION |  |
|  |  | **Region I (Ilocos)** | PANGASINAN |  |
|  |  | **Region II (Cagayan Valley)** | --- |  |
|  |  | **Region II (Cagayan Valley)** | BATANES |  |
|  |  | **Region II (Cagayan Valley)** | CAGAYAN |  |
|  |  | **Region II (Cagayan Valley)** | CITY OF SANTIAGO | ICC |
|  |  | **Region II (Cagayan Valley)** | ISABELA |  |
|  |  | **Region II (Cagayan Valley)** | NUEVA VIZCAYA |  |
|  |  | **Region II (Cagayan Valley)** | QUIRINO |  |
|  |  | **Region III (Central Luzon)** | --- |  |
|  |  | **Region III (Central Luzon)** | ANGELES CITY | HUC |
|  |  | **Region III (Central Luzon)** | AURORA |  |
|  |  | **Region III (Central Luzon)** | BATAAN |  |
|  |  | **Region III (Central Luzon)** | BULACAN |  |
|  |  | **Region III (Central Luzon)** | NUEVA ECIJA |  |
|  |  | **Region III (Central Luzon)** | OLONGAPO CITY | HUC |
|  |  | **Region III (Central Luzon)** | PAMPANGA |  |
|  |  | **Region III (Central Luzon)** | TARLAC |  |
|  |  | **Region III (Central Luzon)** | ZAMBALES |  |
|  |  | **Region V (Bicol)** | **---** |  |
|  |  | **Region V (Bicol)** | ALBAY |  |
|  |  | **Region V (Bicol)** | CAMARINES NORTE |  |
|  |  | **Region V (Bicol)** | CAMARINES SUR |  |
|  |  | **Region V (Bicol)** | CATANDUANES |  |
|  |  | **Region V (Bicol)** | MASBATE |  |
|  |  | **Region V (Bicol)** | NAGA CITY | ICC |
|  |  | **Region V (Bicol)** | SORSOGON |  |
|  |  | **Region VI (Western Visayas)** | --- |  |
|  |  | **Region VI (Western Visayas)** | AKLAN |  |
|  |  | **Region VI (Western Visayas)** | ANTIQUE |  |
|  |  | **Region VI (Western Visayas)** | BACOLOD CITY | HUC |
|  |  | **Region VI (Western Visayas)** | CAPIZ |  |
|  |  | **Region VI (Western Visayas)** | GUIMARAS |  |
|  |  | **Region VI (Western Visayas)** | ILOILO |  |
|  |  | **Region VI (Western Visayas)** | ILOILO CITY | HUC |
|  |  | **Region VI (Western Visayas)** | NEGROS OCCIDENTAL |  |
|  |  | **Region VII (Central Visayas)** | --- |  |
|  |  | **Region VII (Central Visayas)** | BOHOL |  |
|  |  | **Region VII (Central Visayas)** | CEBU |  |
|  |  | **Region VII (Central Visayas)** | CEBU CITY | HUC |
|  |  | **Region VII (Central Visayas)** | LAPU-LAPU CITY (OPON) | HUC |
|  |  | **Region VII (Central Visayas)** | MANDAUE CITY | HUC |
|  |  | **Region VII (Central Visayas)** | NEGROS ORIENTAL |  |
|  |  | **Region VII (Central Visayas)** | SIQUIJOR |  |
|  |  | **Region VIII (Eastern Visayas)** | --- |  |
|  |  | **Region VIII (Eastern Visayas)** | BILIRAN |  |
|  |  | **Region VIII (Eastern Visayas)** | EASTERN SAMAR |  |
|  |  | **Region VIII (Eastern Visayas)** | LEYTE |  |
|  |  | **Region VIII (Eastern Visayas)** | NORTHERN SAMAR |  |
|  |  | **Region VIII (Eastern Visayas)** | ORMOC CITY | ICC |
|  |  | **Region VIII (Eastern Visayas)** | SAMAR (WESTERN SAMAR) |  |
|  |  | **Region VIII (Eastern Visayas)** | SOUTHERN LEYTE |  |
|  |  | **Region VIII (Eastern Visayas)** | TACLOBAN CITY | HUC |
|  |  | **Region IX (Zamboanga)** | --- |  |
|  |  | **Region IX (Zamboanga)** | CITY OF ISABELA | CC |
|  |  | **Region IX (Zamboanga)** | ZAMBOANGA CITY | HUC |
|  |  | **Region IX (Zamboanga)** | ZAMBOANGA DEL NORTE |  |
|  |  | **Region IX (Zamboanga)** | ZAMBOANGA DEL SUR |  |
|  |  | **Region IX (Zamboanga)** | ZAMBOANGA SIBUGAY |  |
|  |  | **Region X (Northern Mindanao)** | --- |  |
|  |  | **Region X (Northern Mindanao)** | BUKIDNON |  |
|  |  | **Region X (Northern Mindanao)** | CAGAYAN DE ORO CITY | HUC |
|  |  | **Region X (Northern Mindanao)** | CAMIGUIN |  |
|  |  | **Region X (Northern Mindanao)** | ILIGAN CITY | HUC |
|  |  | **Region X (Northern Mindanao)** | LANAO DEL NORTE |  |
|  |  | **Region X (Northern Mindanao)** | MISAMIS OCCIDENTAL |  |
|  |  | **Region X (Northern Mindanao)** | MISAMIS ORIENTAL |  |
|  |  | **Region XI (Davao)** | --- |  |
|  |  | **Region XI (Davao)** | DAVAO DE ORO |  |
|  |  | **Region XI (Davao)** | DAVAO CITY | HUC |
|  |  | **Region XI (Davao)** | DAVAO DEL NORTE |  |
|  |  | **Region XI (Davao)** | DAVAO DEL SUR |  |
|  |  | **Region XI (Davao)** | DAVAO OCCIDENTAL |  |
|  |  | **Region XI (Davao)** | DAVAO ORIENTAL |  |
|  |  | **Region XII (SOCCSKSARGEN)** | --- |  |
|  |  | **Region XII (SOCCSKSARGEN)** | COTABATO (NORTH COTABATO) |  |
|  |  | **Region XII (SOCCSKSARGEN)** | GENERAL SANTOS CITY (DADIANGAS) | HUC |
|  |  | **Region XII (SOCCSKSARGEN)** | SARANGANI |  |
|  |  | **Region XII (SOCCSKSARGEN)** | SOUTH COTABATO |  |
|  |  | **Region XII (SOCCSKSARGEN)** | SULTAN KUDARAT |  |
|  |  | **Region IV-A (CALABARZON)** | --- |  |
|  |  | **Region IV-A (CALABARZON)** | BATANGAS |  |
|  |  | **Region IV-A (CALABARZON)** | CAVITE |  |
|  |  | **Region IV-A (CALABARZON)** | LAGUNA |  |
|  |  | **Region IV-A (CALABARZON)** | LUCENA CITY | HUC |
|  |  | **Region IV-A (CALABARZON)** | QUEZON |  |
|  |  | **Region IV-A (CALABARZON)** | RIZAL |  |
|  |  | **Region IV-B (MIMAROPA)** | --- |  |
|  |  | **Region IV-B (MIMAROPA)** | MARINDUQUE |  |
|  |  | **Region IV-B (MIMAROPA)** | OCCIDENTAL MINDORO |  |
|  |  | **Region IV-B (MIMAROPA)** | ORIENTAL MINDORO |  |
|  |  | **Region IV-B (MIMAROPA)** | PALAWAN |  |
|  |  | **Region IV-B (MIMAROPA)** | PUERTO PRINCESA CITY | HUC |
|  |  | **Region IV-B (MIMAROPA)** | ROMBLON |  |
|  |  | **BARMM (Bangsamoro)** | --- |  |
|  |  | **BARMM (Bangsamoro)** | BASILAN |  |
|  |  | **BARMM (Bangsamoro)** | COTABATO CITY | ICC |
|  |  | **BARMM (Bangsamoro)** | LANAO DEL SUR |  |
|  |  | **BARMM (Bangsamoro)** | MAGUINDANAO |  |
|  |  | **BARMM (Bangsamoro)** | SULU |  |
|  |  | **BARMM (Bangsamoro)** | TAWI-TAWI |  |
|  |  | **CAR** | --- |  |
|  |  | **CAR** | ABRA |  |
|  |  | **CAR** | APAYAO |  |
|  |  | **CAR** | BAGUIO CITY | HUC |
|  |  | **CAR** | BENGUET |  |
|  |  | **CAR** | IFUGAO |  |
|  |  | **CAR** | KALINGA |  |
|  |  | **CAR** | MOUNTAIN PROVINCE |  |
|  |  | **CARAGA** | --- |  |
|  |  | **CARAGA** | AGUSAN DEL NORTE |  |
|  |  | **CARAGA** | AGUSAN DEL SUR |  |
|  |  | **CARAGA** | BUTUAN CITY | HUC |
|  |  | **CARAGA** | DINAGAT ISLANDS |  |
|  |  | **CARAGA** | SURIGAO DEL NORTE |  |
|  |  | **CARAGA** | SURIGAO DEL SUR |  |
|  |  | **NCR** | --- |  |
|  |  | **NCR** | CALOOCAN CITY | HUC |
|  |  | **NCR** | CITY OF LAS PIÑAS | HUC |
|  |  | **NCR** | CITY OF MAKATI | HUC |
|  |  | **NCR** | CITY OF MALABON | HUC |
|  |  | **NCR** | CITY OF MANDALUYONG | HUC |
|  |  | **NCR** | CITY OF MANILA | HUC |
|  |  | **NCR** | CITY OF MARIKINA | HUC |
|  |  | **NCR** | CITY OF MUNTINLUPA | HUC |
|  |  | **NCR** | CITY OF NAVOTAS | HUC |
|  |  | **NCR** | CITY OF PARAÑAQUE | HUC |
|  |  | **NCR** | CITY OF PASIG | HUC |
|  |  | **NCR** | CITY OF SAN JUAN | HUC |
|  |  | **NCR** | CITY OF VALENZUELA | HUC |
|  |  | **NCR** | PASAY CITY | HUC |
|  |  | **NCR** | PATEROS |  |
|  |  | **NCR** | QUEZON CITY | HUC |
|  |  | **NCR** | TAGUIG CITY | HUC |

|  | ***NATIONAL LEVEL ONLY -- CONFIRM REGIONAL/PROVINCIAL DATA WITH RESU/CESU/PESUs*** | | | | | | | | | | | | | | |
| --- | --- | --- | --- | --- | --- | --- | --- | --- | --- | --- | --- | --- | --- | --- | --- |
| **4: Epidemic curve by data source: weekly case counts by age groups.** | | | | | | |  |  |  |  |  |  |  |  |  |
| ***Instructions:*** | |  |  |  |  |  |  |  |  |  |  |  |  |  |  |
| 1. Report data using the format below for each surveillance system between July 2021 (or start of system if after this date) through March 2023. | | | | | | | | | | | |  |  |  |  |
| 2. Report zero cases if no cases reported for a particular province/region during indicated time period | | | | | | | | |  |  |  |  |  |  |  |
| 3. Note differing age groups for SARI & RSV surveillance | | | | | |  |  |  |  |  |  |  |  |  |  |
| 4. For SARI sentinel data, breaking into 2 tables; 1 with data by age groups/epi weeks for sites that overlap with RSV pilot sites.  The other with data by age group/epi weeks for non-RSV pilot sites. | | | | | | | | | | | | | | | |
| **COVID-19 Case Reporting** | | |  |  |  |  |  |  |  |  |  |  |  |  |  |
| **Year** | **Epi Week** | **Week** | **Age group 1: 0-4 years** | **Age group 2: 5-11 years** | **Age group 3: 12-17 years** | **Age group 4: 18-29 years** | **Age group 5: 30-39 years** | **Age group 6: 40-49 years** | **Age group 7: 50-59 years** | **Age group 8: 60-69 years** | **Age group 9: 70+ years** | **Missing Age** |  |  |  |
|  |  |  |  |  |  |  |  |  |  |  |  |  |  |  |  |
| **ILI Surveillance** | |  |  |  |  |  |  |  |  |  |  |  |  |  |  |
| **Year** | **Epi Week** | **Week** | **Age group 1: 0-4 years** | **Age group 2: 5-11 years** | **Age group 3: 12-17 years** | **Age group 4: 18-29 years** | **Age group 5: 30-39 years** | **Age group 6: 40-49 years** | **Age group 7: 50-59 years** | **Age group 8: 60-69 years** | **Age group 9: 70+ years** | **Missing Age** |  |  |  |
|  |  |  |  |  |  |  |  |  |  |  |  |  |  |  |  |
| **ILI Sentinel Surveillance** | | |  |  |  |  |  |  |  |  |  |  |  |  |  |
| **Year** | **Epi Week** | **Week** | **Age group 1: 0-4 years** | **Age group 2: 5-11 years** | **Age group 3: 12-17 years** | **Age group 4: 18-29 years** | **Age group 5: 30-39 years** | **Age group 6: 40-49 years** | **Age group 7: 50-59 years** | **Age group 8: 60-69 years** | **Age group 9: 70+ years** | **Missing Age** |  |  |  |
|  |  |  |  |  |  |  |  |  |  |  |  |  |  |  |  |
| **SARI Surveillance** | |  |  |  |  |  |  |  |  |  |  |  |  |  |  |
| **Year** | **Epi Week** | **Week** | **Age group 1: 0 -<1 years** | **Age group 2:  1 - <2 years** | **Age group 1: 2-4 years** | **Age group 2: 5-11 years** | **Age group 3: 12-17 years** | **Age group 4: 18-29 years** | **Age group 5: 30-39 years** | **Age group 6: 40-49 years** | **Age group 7: 50-59 years** | **Age group 8: 60-69 years** | **Age group 9: 70+ years** | **Missing Age** |  |
|  |  |  |  |  |  |  |  |  |  |  |  |  |  |  |  |
| **SARI Sentinel Surveillance** | | |  |  |  |  |  |  |  |  |  |  |  |  |  |
| **Year** | **Epi Week** | **Week** | **Age group 1: 0 -<1 years** | **Age group 2:  1 - <2 years** | **Age group 1: 2-4 years** | **Age group 2: 5-11 years** | **Age group 3: 12-17 years** | **Age group 4: 18-29 years** | **Age group 5: 30-39 years** | **Age group 6: 40-49 years** | **Age group 7: 50-59 years** | **Age group 8: 60-69 years** | **Age group 9: 70+ years** | **Missing Age** |  |
|  |  |  |  |  |  |  |  |  |  |  |  |  |  |  |  |
| **RSV Surveillance Pilot** | | |  |  |  |  |  |  |  |  |  |  |  |  |  |
| **Year** | **Epi Week** | **Week** | **Age group 1: 0 -<1 years** | **Age group 2:  1 - <2 years** | **Missing Age** |  |  |  |  |  |  |  |  |  |  |
|  |  |  |  |  |  |  |  |  |  |  |  |  |  |  |  |
