# Supplementary material for: Optimizing surveillance post-pandemic: an evaluation of COVID-19 and other respiratory virus surveillance systems in the Philippines, April 2023
Source: BMC Public Health. 2025 Oct 8;25:3378. doi: 10.1186/s12889-025-24208-8 (PMC12506167; doi:10.1186/s12889-025-24208-8)
Supplement: Supplementary file 4 — Additional file 4. Additional Table 1: List of evaluation sites visited by region — the Philippines, April 2023. Additional Table 2: Completeness of reporting for respiratory virus surveillance systems at national and sub-national levels during February 26 - April 1, 2023. [file 12889_2025_24208_MOESM4_ESM.docx]

**Additional Table 1: List of evaluation sites visited by region — the Philippines, April 2023**

| **Region** | **Office** | **Hospital** | **Health Office** | **Health Center/Unit** |
| --- | --- | --- | --- | --- |
| **National Capital Region (NCR)** | -Disease Prevention and Control Bureau  Epidemiology Bureau  - Research Institute for Tropical Medicine (RITM)  - Ninoy Aquino International Airport (NAIA) Terminal 1  - Knowledge Management and Information Technology Service (KMITS)  - Bureau of Quarantine | **Public hospital:**  - Lung Center of the Philippines†  - Dr. Jose N. Rodriguez Medical Center*  - Quezon City General Hospital*  - San Lazaro Hospital†§  **Private hospital:**  - St. Luke’s Medical Center (Quezon City) | Quezon City Health Office and Molecular Laboratory | Kamuning Health Center |
| **Cordillera Administrative Region (CAR)** | Regional Epidemiology and Surveillance Unit (Center for Health Development) | - Baguio General Hospital and Medical Center*†  - Benguet General Hospital | - Baguio City Health Office  - Benguet Provincial Epidemiology and Surveillance Unit  - La Trinidad Municipal Health Office | Lucban Health Center* |
| **Eastern Visayas (EV)** | Regional Epidemiology and Surveillance Unit | Eastern Visayas Regional Medical Center | - Leyte Provincial Health Office  - Tacloban City Health Office | - Palo Rural Health Unit  - Kawayan District Health |

*Denotes an ILI sentinel site; †Denotes a SARI sentinel site; §Denotes an RSV sentinel site

**Additional Table 2: Completeness of reporting for respiratory virus surveillance systems at national and sub-national levels during February 26 - April 1, 2023**

| **Surveillance System^1^** | **Number of reporting sites** | **Sites reporting in the past 30 days (%)** |
| --- | --- | --- |
| **COVID-19 Case-based Surveillance** | 374 | 68.5% |
| **ILI Sentinel Surveillance** | 17 | 58.8% |
| **SARI Sentinel Surveillance** | 5 | 76.0% |
| **RSV Sentinel Surveillance** | 3 | 100% |

**^1^** We were unable to assess reporting completeness for traveler screening surveillance and ILI/SARI non-sentinel surveillance

ILI: Influenza-like Illness, SARI: Severe Acute Respiratory Illness, RSV: Respiratory Syncytial Virus
